# Variable cMyBP-C expression from cell to cell in a *MYBPC3*^c.927–2 A>G^ hiPSC-CM model recapitulates HCM patient phenotype

**DOI:** 10.1186/s13287-026-05063-9

**Published:** 2026-05-19

**Authors:** Karina Ivaskevica, Kathrin Kowalski, Birgit Piep, Jana Teske, Joachim D. Meissner, Maike Kosanke, Ante Radocaj, Judith Montag, Robert Zweigerdt, Theresia Kraft, Sarah A. Konze

**Affiliations:** 1 Institute of Molecular and Cell Physiology, Hannover Medical School (MHH), Hannover, Germany; 2 Leibniz Research Laboratories for Biotechnology and Artificial Organs (LEBAO), Clinic for Cardiac, Thoracic, Transplantation and Vascular Surgery, MHH, Hannover, Germany; 3 Research Core Unit Genomics, Hannover Medical School (MHH), Hannover, Germany; 4 Medical School Berlin (MSB), Berlin, Germany

**Keywords:** hiPSC-derived cardiomyocytes, *MYBPC3* mutation, Hypertrophic cardiomyopathy, cMyBP-C expression, Disease modeling, Burst-like transcription

## Abstract

**Background:**

Hypertrophic cardiomyopathy (HCM) is frequently associated with mutations in cardiac myosin binding protein C (cMyBP-C; *MYBPC3*) and cMyBP-C haploinsufficiency. Previously we discovered burst-like transcription of *MYBPC3* and unequal amounts of wild type cMyBP-C from cardiomyocyte to cardiomyocyte in HCM-patient’s myocardium. The present study introduces human induced pluripotent stem cell-derived cardiomyocytes (hiPSC-CMs) carrying the patient-specific heterozygous *MYBPC3* c.927–2 A > G mutation and the respective isogenic control to examine in long-term culture whether comparable pathophysiological features exist in vitro.

**Methods:**

We generated a human induced pluripotent stem cell-derived cardiomyocyte (hiPSC-CM) model harboring the patient-specific *MYBPC3* c.927–2 A > G splice-site mutation. An isogenic control line was used for direct comparison. We assessed cMyBP-C protein expression, transcriptional dynamics, contractile function, and calcium handling, and compared the cellular phenotype to heart tissue from the HCM patient with the same mutation.

**Results:**

cMyBP-C haploinsufficiency in *MYBPC3*^c.927–2 A> G^*-*hiPSC-CMs was confirmed by Western blot. Immunostaining showed myofibrillar disarray and an increasing proportion of cMyBP-C-negative CMs over time for mutant hiPSC-CMs, closely mirrored the variable cMyBP-C protein expression observed in HCM-patient’s myocardium. RNA-FISH revealed variable MYBPC3 transcription from cell to cell, likely contributing to cMyBP-C expression heterogeneity. Twitch shortening velocity slowed over time while Ca²⁺ transient kinetics accelerated in mutant hiPSC-CMs. Transcriptomic analysis revealed dysregulation of pathways associated with contraction, calcium handling, and HCM.

**Conclusions:**

This study presents a validated hiPSC-based model of *MYBPC3*-associated HCM that captures the variability in protein expression and functional phenotype observed in patient heart tissue. Our findings support the relevance of single-cell transcriptional variability in HCM pathogenesis and highlight the utility of this model for future studies.

**Supplementary Information:**

The online version contains supplementary material available at 10.1186/s13287-026-05063-9.

## Introduction

Hypertrophic cardiomyopathy (HCM) is an inherited myocardial disease with an estimated prevalence of 1:500 to 1:200 [1, 2]. HCM is characterized by left ventricular hypertrophy, diastolic dysfunction, and an elevated risk of sudden cardiac death [1]. The main histopathological hallmarks of HCM are cellular and myofibrillar disarray, and interstitial fibrosis [1]. Approximately half of HCM patients are diagnosed with a mutation in a sarcomeric gene, nearly all of which are heterozygous. Notably, depending on the cohort analyzed, up 40% of all HCM-associated sarcomeric mutations occur in cardiac myosin-binding protein C (cMyBP-C), which is encoded by the *MYBPC3* gene [3, 4].

cMyBP-C is a thick filament-associated protein, localized in the cross-bridge bearing C-zone of the A band within the sarcomere [5, 6]. Recent cryo-EM studies suggest that the C-terminal region of cMyBP-C associates with the myosin tail along the thick-filament backbone, whereas titin may indirectly influence cMyBP-C positioning. In contrast, the N-terminal region is proposed to extend away from the thick filament toward the interfilament space, where it may engage myosin subfragment-2 (S2) and/or the regulatory light chain, and has also been suggested to interact with actin and other thin-filament proteins [7–12]. Functionally, cMyBP-C seems to modulate both, the strength and speed of cardiac muscle contraction [13].

HCM mutations in *MYBPC3* are known to present with variable phenotypes, ranging from mild to severe cases [14, 15]. Most mutations in the *MYBPC3* gene lead to premature stop codons and result in degradation of the mutated mRNA or protein. This often causes haploinsufficiency i.e. reduced levels of functional wild type full-length cMyBP-C protein [3]. Recent studies have shown that *MYBPC3* expression of the two alleles is not continuous but occurs in bursts, a phenomenon known as burst-like transcription [16]. Stochastic and independent transcription of the two alleles has minimal impact on cMyBP-C protein levels in healthy heart tissue. However, in HCM heart tissue, unequal expression of mutant and wild type *MYBPC3*-alleles across cardiomyocytes (CMs) results in variable levels of wild type mRNA and protein from CM to CM. This was indeed observed for cardiac tissue of HCM patients with heterozygous *MYBPC3-*mutations [16]. Furthermore, in functional studies it was shown that CMs isolated from the same heart tissue sections vary significantly in calcium sensitivity and force generation [16]. This functional variability suggests an unbalanced contraction between individual CMs within the myocardium of the patients. Notably, truncated cMyBP-C protein fragments could not be detected, suggesting their degradation by nonsense-mediated decay [17].

In this study, we aimed to find out whether unequal expression of wild type cMyBP-C protein among individual CMs observed in HCM-patient cardiac tissue is also present in human-induced pluripotent stem cell-derived cardiomyocytes (hiPSC-CMs). Such CMs likely represent a model for an early stage of the disease and would allow us to examine mechanisms of development and effect of variable cMyBP-C expression in HCM. This cellular model could also serve as a platform for studying and modulating burst-like transcription in HCM.

We introduced a heterozygous truncating mutation in *MYBPC3* (c.927–2 A > G) into an established healthy donor hiPSC line and compared the mutant hiPSC-CMs with the isogenic control. The c.927–2 A > G mutation in *MYBPC3* is identical to the mutation found in patient H84 [16], diagnosed with NYHA class IV heart failure, who underwent heart transplantation at the age of 45. The intronic c.927–2 A > G mutation leads to incorrect splicing and thus introduces a premature stop codon between domains C1 and C2 in the N-terminal region, resulting in cMyBP-C haploinsufficiency.

## Methods

### Cell lines

Mutation c.927–2 A > G was introduced into the hiPSC line HSC ADCF SeV-iPS2 MHHi001-A (https://hpscreg.eu/cell-line/MHHi001-A), by Applied StemCell Inc. using the CRISPR/Cas9 technique. In addition to two heterozygous clones, one isogenic control clone, which had undergone the mutagenesis procedure but only received a silent mutation in the PAM site, was obtained. The heterozygous mutation was confirmed by Sanger sequencing (Supplemental Fig. 1A-B). Cells were routinely screened for Mycoplasma using the Mycoplasma Detection Kit (InvivoGen). Karyotype analysis was performed at the Institute of Human Genetics, Hannover Medical School, and revealed a normal female karyotype (46, XX) for the isogenic control clone, heterozygous clone 1, and heterozygous clone 2 (Supplemental Fig. 1C). Pluripotency was assessed by flow cytometry using antibodies against the pluripotency markers NANOG, OCT-3/4, SSEA-4 and TRA-1-60, demonstrating high purity of pluripotent cells (Supplemental Fig. 2). Genomic stability was evaluated by detection of recurrent genetic abnormalities using the iCS-digital™ PSC test (Stem Genomics), as previously described [20] (Supplemental Fig. 3).

### Differentiation of hiPSC-CMs

hiPSC differentiation into cardiomyocytes (hiPSC-CMs) was performed in suspension culture as described [19–21]. Briefly, hiPSCs were inoculated in Erlenmeyer flasks in mTeSR medium (Thermo Fisher Scientific), supplemented with Rho kinase inhibitor Y-27,632 (RI, STEMCELL Technologies). After two days of aggregate formation, cardiomyogenic differentiation was chemically induced with 5 µM CHIR99021 (STEMCELL Technologies) in CDM3 medium (RPMI 1640 Medium, HEPES (Thermo Fisher Scientific); supplemented with 0.5 mg/mL recombinant human serum albumin (Sigma-Aldrich; A0237) and 213 µg/mL L-ascorbic acid (Merck). After 24 h, the medium was replaced with CDM3 supplemented with 2 µM WNT pathway inhibitor Wntc-59, and the cells were cultured for an additional 48 h. On day 3 of differentiation, the medium was fully exchanged for plain CDM3, followed by 75% medium exchanges every two days. By day 10, the efficiency of cardiomyogenic differentiation was assessed via flow cytometry of dissociated cardiac bodies for the expression of cardiac-specific markers cTnT, MF20, cMyBP-C, NkX2.5, and β-myosin revealing > 95% of cells positive for these markers, indicating high hiPSC-CMs content.

### Cultivation of hiPSC-CMs

Cardiac bodies were dissociated into single cardiomyocytes using the STEMdiff™ Cardiomyocyte Dissociation Kit (STEMCELL Technologies) according to manufacturer’s instructions (5 min mixing at 1000 rpm, 37 °C). Dissociated cardiomyocytes were resuspended in isolation medium composed of IMDM GlutaMAX™ (Gibco, Thermo Fisher Scientific), supplemented with 10% fetal calf serum (FCS; Cytiva, formerly GE Healthcare Life Sciences), 1 mM L-glutamine (Thermo Fisher Scientific), 1× MEM non-essential amino acids (NEAA; Thermo Fisher Scientific), 0.1 mM β-mercaptoethanol (Thermo Fisher Scientific), 100 U/ml penicillin-streptomycin (Thermo Fisher Scientific), and 10 µM ROCK inhibitor Y-27,632 (STEMCELL Technologies) and were plated onto laminin-coated glass cover slips (20 µg/ml laminin; Roche). For Western blot analysis and mRNA sequencing, 100,000 cells were seeded per cover slip. For immunofluorescent staining, 8,000 cells per cover slip were used. For twitch measurements, calcium transients, and FISH analysis, 8,000–10,000 cells were seeded per cover slip. For morphological assessments and analysis of cMyBP-C protein distribution, individual cardiac bodies were manually transferred onto nanopatterned glass substrates (ANFS CS 25 NanoSurface Plates; Curi Bio). After 24 h, the isolation medium was replaced with RPMI 1640 GlutaMAX™ (Gibco, Thermo Fisher Scientific) supplemented with B-27™ Supplement (Thermo Fisher Scientific) and 100 U/ml penicillin-streptomycin. Cells were cultivated for designated time points (day 35, day 56, and days 70–80, referred to as 70+) with medium changes every 2–3 days. Time in culture was counted from the day of plating (day 0). Based on previous findings that replating alters cardiomyocyte maturation programs [22], all experiments were performed on continuously cultured cells without replating.

### Western Blot

For cMyBP-C protein analysis, hiPSC-CMs were cultured on laminin-coated glass cover slips (20 µg/ml laminin; Roche, Thermo Fisher Scientific) for 35, 56, and 70 + days. At each designated time point, cover slips were collected and stored at -80 °C until further processing. For Western blot analysis, cells were lysed in kinase buffer consisting of 20 mM Tris-acetate (pH 7.0), 0.1 mM EDTA, 1 mM EGTA, 1 mM Na₃VO₄, 10 mM β-glycerophosphate, 50 mM NaF, 5 mM sodium pyrophosphate, 0.27 M sucrose, and 1% Triton X-100, supplemented with protease inhibitor cocktail and PhosSTOP phosphatase inhibitor cocktail (Roche). Lysates were mixed with ROTILoad1 loading buffer (Carl Roth) in a 4:1 ratio and denatured at 85 °C for 4 min. Proteins were separated using 4–15% TGX precast SDS-PAGE gels (Criterion™, Bio-Rad) and transferred onto PVDF membranes (0.45 μm pore size; Thermo Fisher Scientific). Membranes were blocked with 3% milk powder (Santa Cruz Biotechnology) in Tris-buffered saline with 0.1% Tween-20 (TBS-T) (Sigma-Aldrich) over night at room temperature. Primary antibodies were diluted in TBS-T and incubated for 2 h at room temperature. The following antibodies were used: anti-cMyBP-C (N-terminal; AB262964, abcam) and anti-α-actinin (A7811, Sigma-Aldrich). After washing, membranes were incubated with HRP-conjugated secondary antibodies (Bio-Rad) in TBS-T for 1 h at room temperature. Protein bands were visualized using the ImageQuant LAS 4000 imaging system (Cytiva) and quantified using ImageQuant TL 1D software (Cytiva). Band intensities were normalized to α-actinin to account for loading differences. To enable cross-gel comparisons, values were further normalized to isogenic control samples run in parallel.

### Immunofluorescent staining and assessment of cMyBP-C mosaic-pattern

To assess the distribution of cMyBP-C in hiPSC-CMs, cells were cultured on nanopatterned glass substrates (ANFS CS25 NanoSurface Plates; Curi Bio) and analyzed at designated time points (days 35, 56, and 70+). Immunofluorescent staining was performed following a standard protocol. Briefly, cells were fixed with 4% paraformaldehyde (PFA; Alfa Aesar) in Dulbecco’s phosphate-buffered saline (DPBS) for 30 min at room temperature. After fixation, cells were washed with DPBS and permeabilized using 0.2% Triton X-100 (Roche) for 15 min, followed by additional DPBS washes. Blocking was performed in 5% bovine serum albumin (BSA; Gerbu Biotechnik GmbH) for 20 min at room temperature to prevent nonspecific antibody binding. Cells were then incubated for 2 h at room temperature with primary antibodies against the N-terminus of cMyBP-C (rabbit polyclonal; AB262964, abcam) and α-actinin (mouse monoclonal clone EA-53; AB9465, abcam). After washing with DPBS, cells were incubated with species-specific secondary antibodies for 1 h at room temperature: Alexa Fluor 488 donkey anti-mouse IgG (A21206; Thermo Fisher Scientific) for α-actinin and Alexa Fluor 555 donkey anti-rabbit IgG (A31570; Thermo Fisher Scientific) for cMyBP-C. Nuclear staining was performed using DAPI (Sigma-Aldrich). All antibody dilutions and staining durations were consistent across experiments. Fluorescence imaging was performed using an Olympus IX83 inverted fluorescence microscope with a 40x objective. Only hiPSC-CMs displaying regular α-actinin striations were included in the analysis. Based on the cMyBP-C staining pattern, cells were categorized into three groups: (1) homogeneous cMyBP-C signal (2), weak or inhomogeneous cMyBP-C signal, and (3) cMyBP-C-negative cells.

### Morphological analysis of hiPSC-CMs

For morphological analysis, hiPSC-CMs cultured on nanopatterned substrates (ANFS CS25 Nanosurface Plate, Curi Bio) and stained for α-actinin and cMyBP-C, as described previously, were used. Cell area and aspect ratio were calculated using ImageJ software (National Institutes of Health). The aspect ratio was determined as the ratio of the maximum cell length to the maximum cell width. Myofibrillar alignment was assessed using the *FibrilTool* plugin for ImageJ [23]. Prior to calculating the alignment scores, the fluorescence images were processed with a bandpass filter to blur sarcomeric striations, ensuring more accurate myofibril alignment score calculation.

### Twitch contraction measurements

Twitch contractions of hiPSC-CMs were recorded using a video-based optical edge-detection contraction analysis system (MyoCam, IonOptix, Milton, MA, USA), as previously described [24, 25]. Briefly, single hiPSC-CMs plated on laminin-coated glass cover slips were placed in a custom-made perfusion chamber. Electrical stimulation was applied using the MyoPacer EP Cell Stimulator (IonOptix Corp.) through two platinum electrodes positioned on either side of the chamber. Cells were stimulated with supra-threshold voltage stimuli (35V) of 4 ms duration at a frequency of 1 Hz. Twitch recordings were initiated only after stable 1 Hz pacing and signal quality were established (1 min after initiating pacing). To minimize time-dependent drift across samples, each coverslip was measured for a maximum of 30 min before proceeding to the next sample. All experiments were performed at a controlled temperature of 37 ± 0.5 °C.

For each cell, 20–30 twitches were averaged for analysis. Key parameters, including time to peak (*ttp*, from baseline to peak shortening), half-relaxation time (*hrt*, time from peak to 50% relaxation), maximum contraction velocity (*V*_*max*_ in µm/s), and contraction amplitude were measured using IonWizard software (IonOptix, Milton, MA, USA).

### Intracellular calcium transient measurements

For intracellular calcium recordings, hiPSC-CMs were loaded with the ratiometric calcium indicator Fura-2 AM (Thermo Fisher Scientific) for 30–40 min. After loading, cells were washed with RPMI 1640 Glutamax culture medium for 10 min and placed in the same perfusion chamber used for twitch contraction analysis. The cells were paced with electrical stimulation at 1 Hz (35 V) under controlled temperature conditions (37 ± 0.5 °C). Recordings were started 1 min after initiation of pacing and only after stable signal quality and 1 Hz pacing were established. To minimize time-dependent drift due to sample deterioration, each coverslip was measured for a maximum of 30 min before proceeding to the next sample.

Calcium transients were recorded by alternating excitation wavelengths at 340 nm and 380 nm, with emission detected at 510 nm. Autofluorescence was measured from 10 to 15 unloaded cells from the same batch and subtracted from the recordings to ensure accurate fluorescence ratio (340/380 nm) calculations. Calcium dynamics, including time to peak (Ca^2+^-*ttp*), half-decay time (Ca^2+^-*hdt*), and calcium amplitude (340/380 nm ratio) were analyzed using IonWizard software (IonOptix Corp.).

### Fluorescence in situ hybridization (FISH) for visualization of active transcription sites (aTS) in hiPSC-CMs

Visualization of aTS was performed as previously described [16]. Briefly, two probe sets were designed using the Stellaris^®^ Probe Designer (https://www.biosearchtech.com/support/tools/design-software/stellaris-probe-designer), to hybridize with intronic and exonic sequence of *MYBPC3* [15]. Exonic sets were labelled with fluorophore Quasar 570 (LGC Biosearch Technologies) and intronic sets were labelled with fluorophore Quasar 670 (LGC Biosearch Technologies). Both, exonic and intronic probe sets contained the standard count of 48 20-mers.

FISH followed the modified protocol of Lyubimova et al. [26]. hiPSC-CMs were fixed for 20 min with 4% PFA (in PBS) at room temperature, followed by three times washing with 1x PBS (w/o Mg^2+^, Ca^2+^). Cells were permeabilized for 1 h in 70% EtOH at 4 °C and washed in wash buffer (10% formamide, 2x saline-sodium citrate (SSC) in nuclease-free water) for 2–5 min. Probes were applied in 50 µl of 125 nmol/L hybridization buffer [26], and hybridized over-night at 37 °C in a sealed humidified chamber. After hybridization, cells were washed twice for 30 min with wash buffer at 37 °C. In the second wash step, DAPI was added at a concentration of 80 ng/mL. Cells were incubated in 2xSSC for 2–5 min at room temperature to remove excessive DAPI solution. 8 µL GLOX anti-fade buffer [26] were added and samples were stored on ice until imaging.

hiPSC-CMs were imaged with an Olympus IX83 fluorescence microscope with a 60x oil objective (ApoN TIRFMN.A. 1.49, Olympus, Tokyo, Japan) and a metal halide light source. Images were recorded with a cooled CCD camera (Orca-R2, Hamamatsu, Photonics, Japan). To analyze nuclei in total, three-dimensional z-stacks were recorded with motorized shutter and z-stage using filter sets for DAPI (Chroma U-F4900, Chroma Technology Corp, Bellows Falls VT, USA), GFP (Chroma U-F49002), Cy3 (Chroma U-F49004) and Cy5 (Chroma U-F49006). Exposure times for DAPI were 15 ms, 150 ms for GFP, 800 ms for Quasar 570/Cy3, and 1 s for Quasar 670/Cy5. Adjacent images in z-stacks were separated by 0.3 μm.

cellSens Dimension Desktop (version 2.3, Olympus, Tokyo, Japan) was used to count the number of active transcription sites (spots with co-localized fluorescence signal for intronic and exonic probe set in cardiomyocyte-nuclei) of single cardiomyocytes, which were verified by cytoplasmic mRNA and/or striation patterns (bright field image). Transcriptional activity was determined by calculation of aTS per nucleus.

### RNA isolation and sequencing

Total RNA was isolated from hiPSC-derived cardiomyocytes (hiPSC-CMs) cultured for 35 and 70 + days using the Monarch Total RNA Miniprep Kit (New England Biolabs), following the manufacturer’s instructions. For day 35, two independent mRNA samples from three separate differentiation batches were analyzed for both the isogenic control and heterozygous mutant lines. For day 70+, one mRNA sample from each of three independent differentiation batches was used for analysis.

### Library generation, quality control, and quantification

200 ng of total RNA per sample were utilized as input for mRNA enrichment procedure with ‘NEBNext^®^ Poly(A) mRNA Magnetic Isolation Module’ (E7490L; New England Biolabs) followed by stranded cDNA library generation using ‘NEBNext^®^ Ultra II Directional RNA Library Prep Kit for Illumina’ (E7760L; New England Biolabs). All steps were performed as recommended in user manualE7760 (Version 1.0_02-2017; NEB) except that all reactions were downscaled to 2/3 of initial volumes.

cDNA libraries were barcoded by dual indexing approach, using ‘NEBNext Multiplex Oligos for Illumina − 96 Unique Dual Index Primer Pairs’ (6440S; New England Biolabs). All generated cDNA libraries were amplified with 9 cycles of final PCR.

One additional purification step was introduced at the end of the standard procedure, using 1.2x ‘Agencourt^®^ AMPure^®^ XP Beads’ (#A63881; Beckman Coulter, Inc.). Fragment length distribution of individual libraries was monitored using ‘Bioanalyzer High Sensitivity DNA Assay’ (5067 − 4626; Agilent Technologies). Quantification of libraries was performed by use of the ‘Qubit^®^ dsDNA HS Assay Kit’ (Q32854; ThermoFisher Scientific).

### Library denaturation and sequencing run

Equal molar amounts of individually barcoded libraries were pooled for a common sequencing run in which each analyzed library constituted around 5.6% of overall flowcell / run capacity. The library pool was denatured with NaOH and was finally diluted to 1.8pM according to the Denature and Dilute Libraries Guide (Document # 15048776 v02; Illumina). 1.3 ml of the denatured pool was loaded on an Illumina NextSeq 550 sequencer using a High Output Flowcell (400 M cluster) for single reads (20024906; Illumina). Sequencing was performed with the following settings: Sequence reads 1 and 2 with 38 bases each; Index reads 1 and 2 with 8 bases each.

### BCL to FASTQ conversion

BCL files were converted to FASTQ files using bcl2fastq Conversion Software version v2.20.0.422 (Illumina).

### Raw data processing and quality control

Raw data processing was conducted by use of nfcore/rnaseq (version 3.9) which is a bioinformatics best-practice analysis pipeline used for RNA sequencing data at the National Genomics Infrastructure at SciLifeLab Stockholm, Sweden. The pipeline uses Nextflow, a bioinformatics workflow tool. It pre-processes raw data from FastQ inputs, aligns the reads and performs extensive quality-control on the results. The genome reference and annotation data were taken from GENCODE.org (Homo sapiens: GRCh38.p13; release 39).

### Normalization and differential expression analysis

Normalization and differential expression analysis was performed on the internal Galaxy (version 20.05) instance of the RCU Genomics, Hannover Medical School, Germany with DESeq2 (Galaxy Tool Version 2.11.40.6) with default settings except for “Output normalized counts table”, which was set to “Yes” and all additional filters were disabled (“Turn off outliers replacement”, “Turn off outliers filtering”, and “Turn off independent filtering” set “Yes”). Analysis was performed with multiple levels of primary factor (all different conditions) comparing all levels against each other.

### RNA-seq data analyses

Enrichr (https://maayanlab.cloud/Enrichr/) was used for identifying the HCM-associated pathways from mRNA sequencing (KEGG database). Data of differently expressed genes for d35 and d70 + are summarized in Supplemental Table 1.

### Statistical analysis

Results are presented as mean ± standard deviation (SD). Comparisons between two groups were performed using the Mann-Whitney U test, as data were not normally distributed. Western blot results were analyzed using an unpaired t-test. Normality was assessed using Shapiro-Wilk test. All statistical analyses were performed using GraphPad Prism version 8.0.1 (GraphPad Software).

## Results

### cMyBP-C expression and *MYBPC3* transcriptional activity

**Fig. 1:**
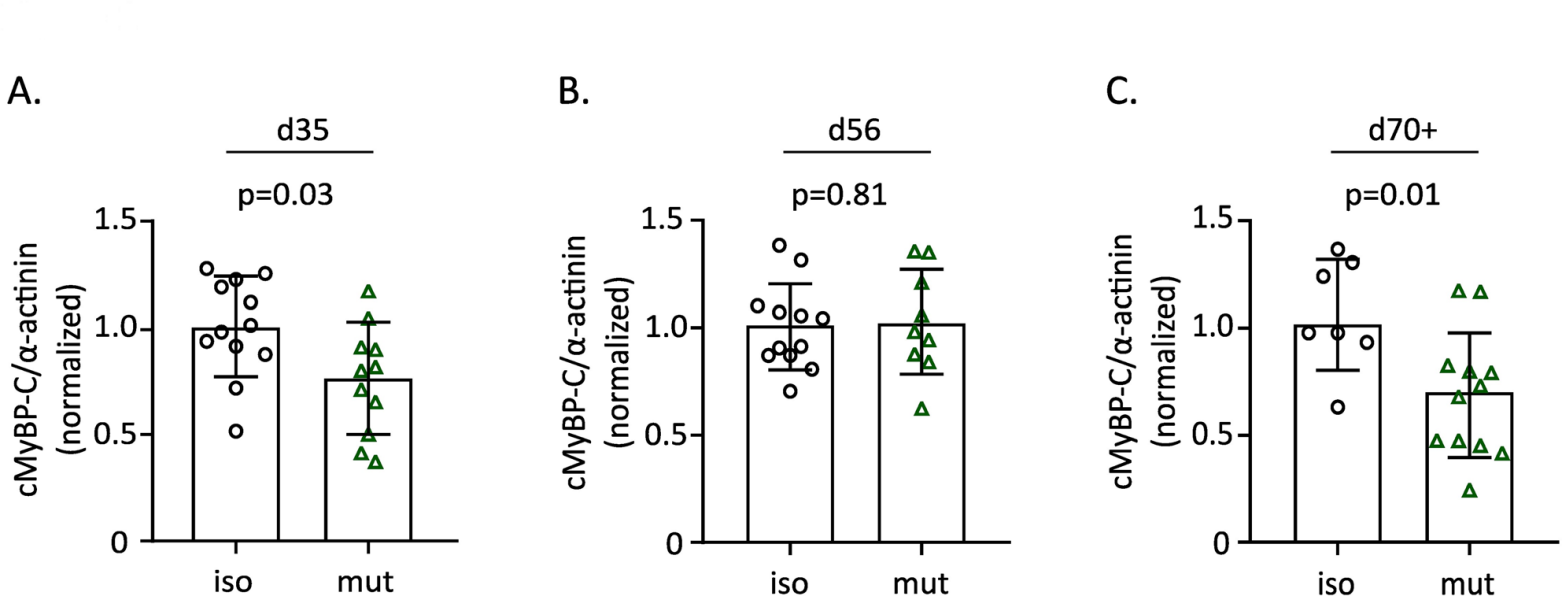
Western Blot analysis of cMyBP-C protein levels in *MYBPC3*^c.927–2 A> G^ hiPSC-CMs and isogenic control. cMyBP-C levels were normalized to α-actinin. The mean value of all cMyBP-C/α-actinin ratios of each time point was set = 1 to enable across gel comparisons. Data are presented as mean ± SD, analyzed from *N* = 3 independent differentiations. Differentiations were performed using one isogenic control clone and one heterozygous *MYBPC3* mutant clone. Statistical comparisons were analyzed using the unpaired t-test

To assess cMyBP-C protein levels longitudinally during hiPSC-CM cultivation, Western blot analysis was performed (Fig. 1). At d35, a ~ 25% reduction in total cMyBP-C levels was observed in the mutant compared to the isogenic control (Fig. 1A), while at d56, cMyBP-C levels in the mutant were comparable to the control (Fig. 1B). However, by d70+ (Fig. 1C), cMyBP-C levels were reduced again, with a ~ 30% reduction. These findings confirm haploinsufficiency in the hiPSC-CM model, aligning with previous studies on patient myocardial tissue with the same mutation.

**Fig. 2:**
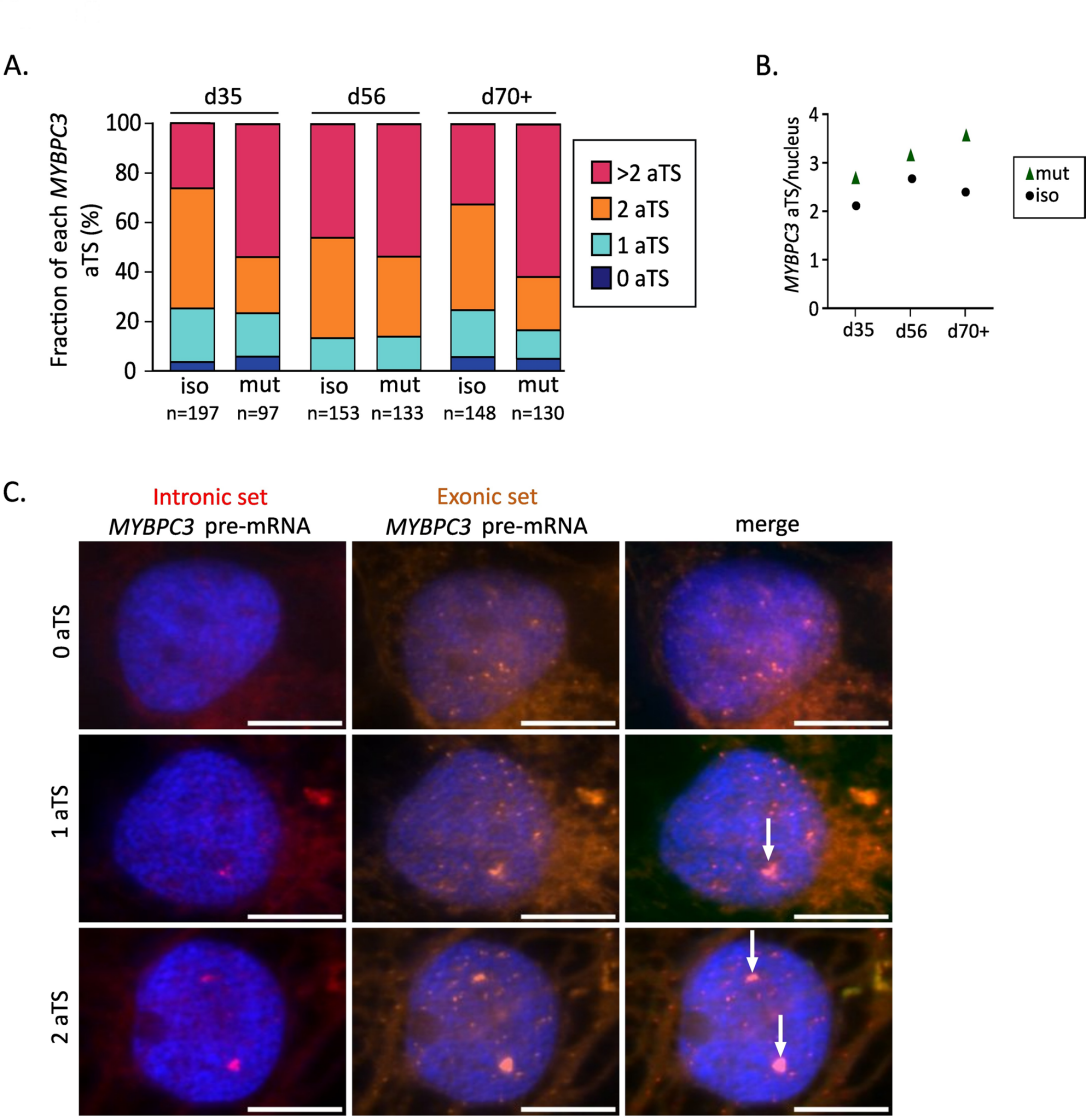
Analysis of active transcription sites (aTS) of *MYBPC3* in *MYBPC3*^c.927–2 A> G^ and isogenic control hiPSC-CMs. **A** Distribution of the numbers of aTS per nucleus in cardiomyocytes for *MYBPC3*. The percentage of nuclei with 0, 1, 2, > 2 aTS was plotted for single cardiomyocytes from *N* = 3 differentiations. Differentiations were performed using one isogenic control clone and one heterozygous *MYBPC3* mutant clone. *n* = number of analyzed nuclei. **B** Average number of aTS per nucleus for *MYBPC3* in isogenic control (black circles) and mutant (green triangles) at d35, d56 and d70 + post differentiation. **C** Representative images of 0 aTS, 1 aTS, and 2 aTS in isogenic control. White arrows indicate aTS within the nucleus. Scale bar 5 μm

Since the *MYBPC3*, *MYH7*, and *TNNI3* genes have been reported to be expressed in bursts [16, 27, 28], we examined whether *MYBPC3* exhibits a similar mode of transcription in our hiPSC-CM model. To visualize actively transcribed alleles, we used single-molecule RNA fluorescence in situ hybridization (RNA-FISH, Fig. 2). Fluorescently labelled probe sets enable the detection of pre-mRNA and spliced mRNA, with the co-localization of these probes indicating active transcription sites (aTS, Fig. 2C).

We found that both the *MYBPC3*^c.927–2 A> G^ mutant hiPSC-CMs and the isogenic control exhibited burst-like *MYBPC3* transcription across all analyzed time points (Fig. 2A). This is evident from nuclei lacking aTS and those with a single aTS. The burst-like transcription pattern was similar between the mutant and the isogenic control CMs. However, over the cultivation period, the number of aTS per nucleus suggested higher transcriptional activity in the *MYBPC3*^c.927–2 A> G^ mutant compared to the isogenic control (Fig. 2B). In detail, at d35, the mutant showed 2.71 aTS/nucleus compared to 2.11 aTS/nucleus in the isogenic control. By d56, these values increased to 3.18 in the mutant and 2.69 in the control. The most pronounced difference occurred at d70+, with the mutant reaching 3.60 aTS/nucleus versus 2.38 in the isogenic control.

**Fig. 3:**
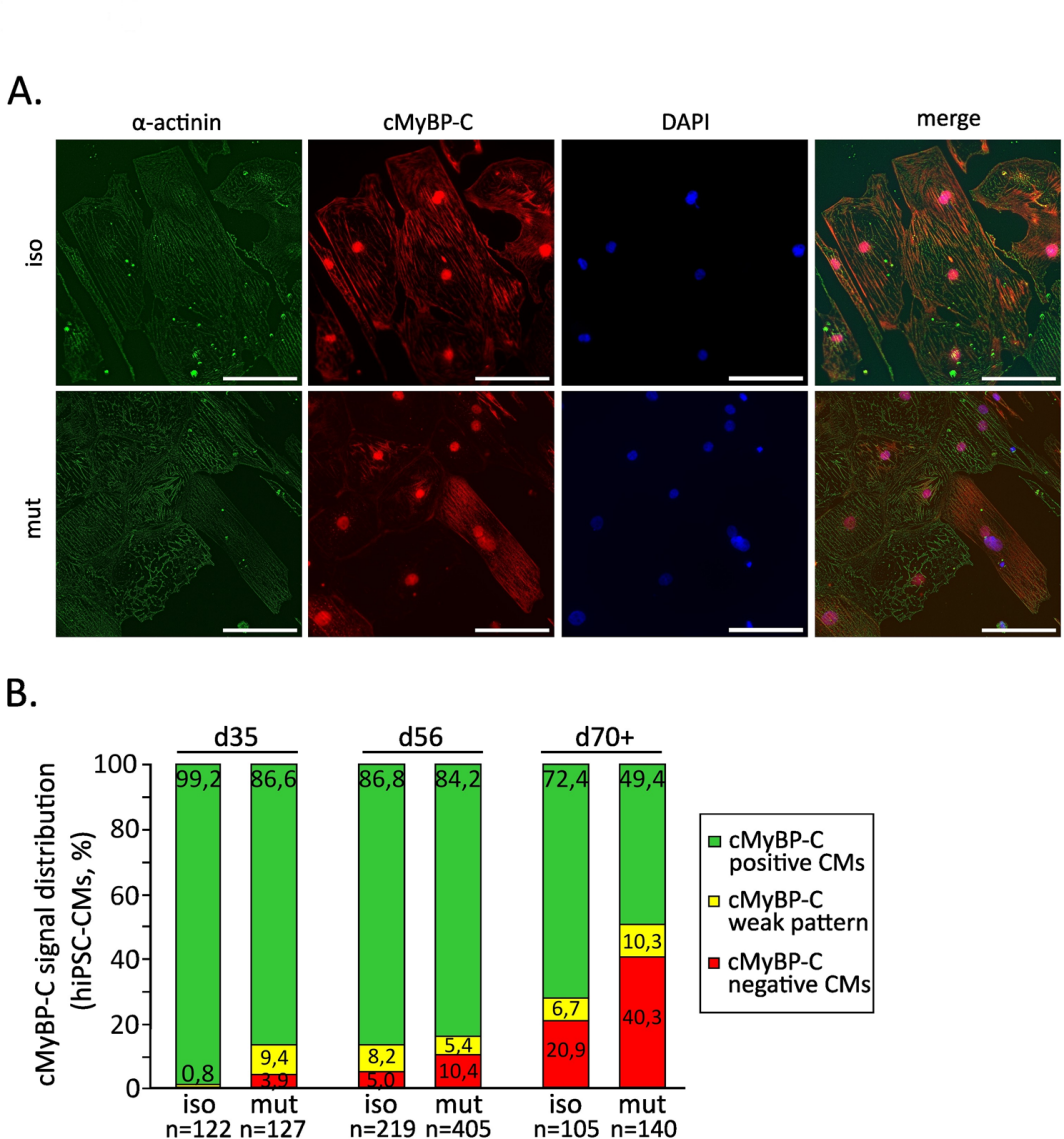
Assessment of variable cMyBP-C protein expression from CM to CM. **A** Immunofluorescent images of isogenic controls and *MYBPC3*^c.927–2 A> G^ hiPSC-CMs after 70 + days of cultivation showing variable sarcomeric expression of cMyBP-C protein from CM to CM. Scale bar 100 μm. **B** Percentage of cMyBP-C-positive CMs in mutant and isogenic controls at d35, d56, and d70+, categorized into three distinct groups: cMyBP-C strong pattern (green), and weak pattern (yellow) stands for weak or inhomogeneous cMyBP-C signal in individual CMs, respectively. cMyBP-C negative CMs indicates lack of cMyBP-C expression (red). Only CMs with clear cell boarders were taken into analysis. *n* = number of analyzed single cells in *N* = 2 differentiations. Differentiations were performed using one isogenic control clone and one heterozygous *MYBPC3* mutant clone

Since *MYBPC3* displayed burst-like transcription in our cellular model, we proceeded to analyze the distribution of cMyBP-C protein among individual CMs using immunofluorescent staining (Fig. 3A). Notably, cMyBP-C-negative CMs were observed in both mutant hiPSC-CMs and isogenic controls; however, the proportion of cMyBP-C negative CMs in the mutant was consistently about twice as high at all analyzed time points (Fig. 3B). At d35, approximately 4% of mutant CMs lacked detectable cMyBP-C protein. This proportion increased to about 10% by d56. By d70+, approximately 40% of mutant CMs lacked cMyBP-C protein, indicating a pronounced increase in the variable distribution of cMyBP-C among individual CMs.

### Morphological characterization and myofibrillar disarray

**Fig. 4:**
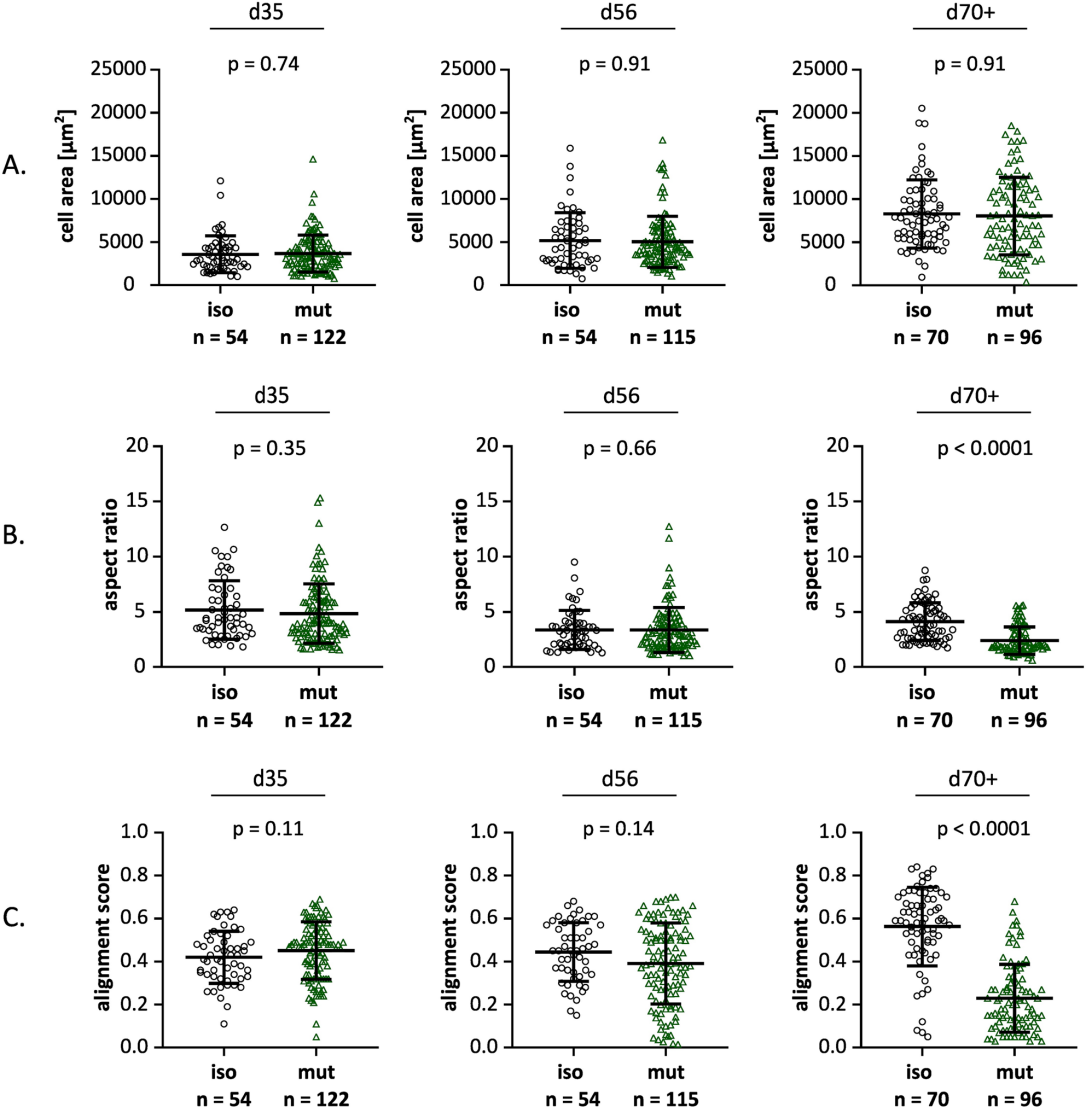
Morphological analysis of *MYBPC3*^c.927–2 A> G^ and isogenic control hiPSC-CMs, cultivated on nanopatterned substrates. Cellular area (**A**), aspect ratio (**B**), and myofibrillar alignment score (**C**) calculated for mutant and isogenic controls hiPSC-CMs using ImageJ. *n* = number of analyzed single cells in *N* = 2 differentiations. Differentiations were performed using one isogenic control clone and one heterozygous *MYBPC3* mutant clone. Data are presented as mean ± SD. Statistical comparisons were analyzed using Mann-Whitney test

hiPSC-CM models for HCM typically exhibit increased cellular area, alongside a decrease in aspect ratio (maximum length/maximum width). Additionally, cardiomyocyte and myofibrillar disarray has been reported in HCM heart tissues [29]. To evaluate whether this phenotype is also present in our hiPSC-CM disease model, we investigated the cellular morphology and myofibrillar alignment of *MYBPC3*^c.927–2 A> G^ and isogenic control hiPSC-CMs (Fig. 4).

Both *MYBPC3*^c.927–2 A> G^ and isogenic control hiPSC-CMs showed an increase in cell area with time of cultivation, but there was no difference between mutant CMs and isogenic controls at all analyzed time points (Fig. 4A). By d70+, the mutant CMs exhibited a higher proportion of roundish-shaped CMs, as evidenced by a reduced aspect ratio (Fig. 4B). The alignment score increased at the latest time point for the isogenic controls, while it was reduced for the mutant CMs, indicating increased myofibrillar disarray in *MYBPC3*^c.927–2 A> G^ hiPSC-CMs (Fig. 4C). Taken together, these findings show that by d70+, the majority of *MYBPC3*^c.927–2 A> G^ hiPSC-CMs did not display an elongated shape with well-aligned myofibrils, which would be characteristic of healthy cardiomyocytes.

### Impact of c.927–2 A > G *MYBPC3* mutation on twitch kinetics and calcium transients

To investigate the impact of c.927–2 A > G *MYBPC3* mutation on contraction kinetics, the isolated hiPSC-CMs were cultivated on laminin-coated glass cover slips for 35, 56, and 70 + days and assessed for twitch kinetics at 1 Hz/35 V, and 37 °C.

**Fig. 5:**
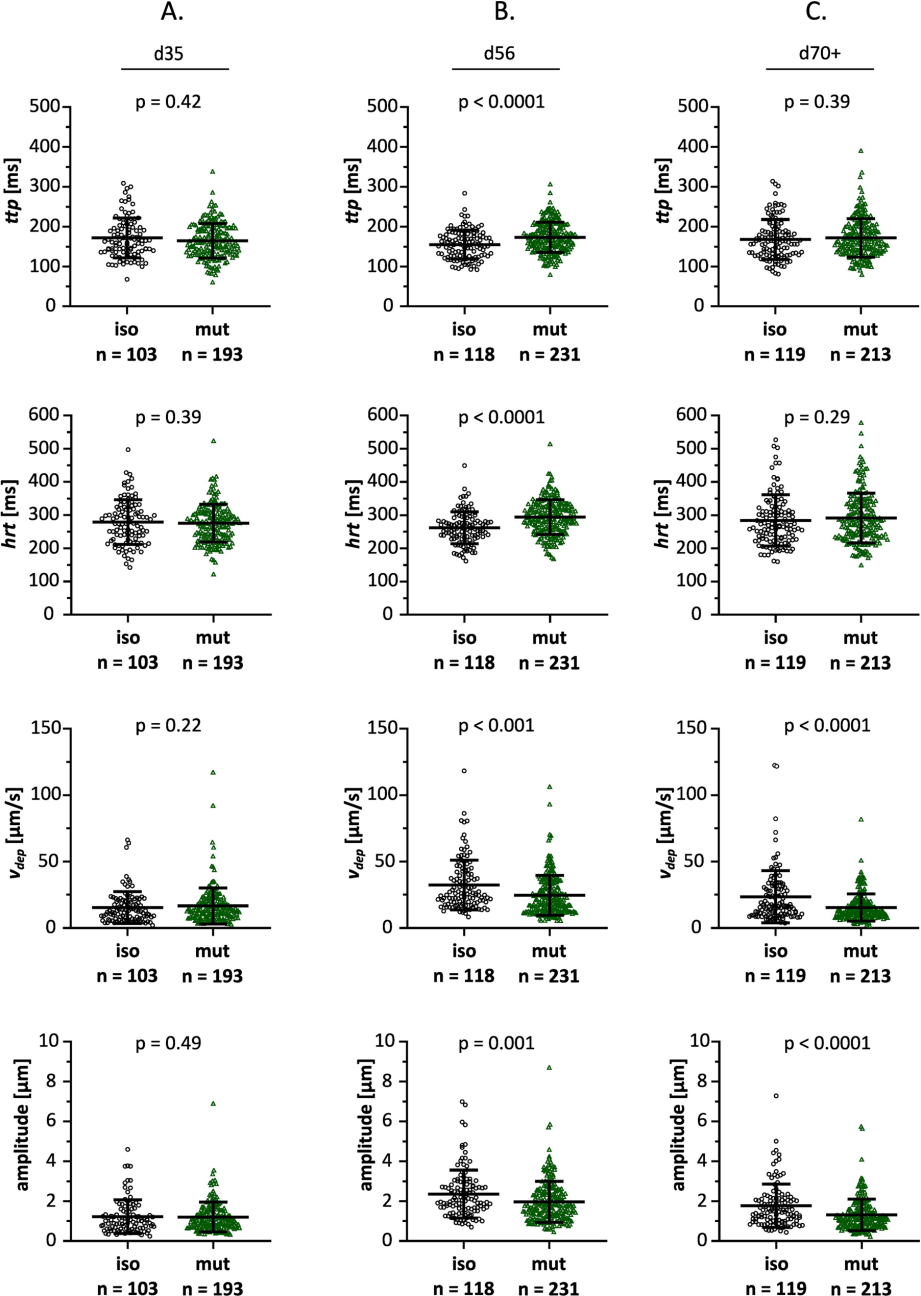
Scatter plots of twitch kinetics parameters of *MYBPC3*^c.927–2 A> G^ and isogenic control hiPSC-CMs cultivation days. Time to peak (*ttp*): the time at which the peak is reached starting from the electrical stimulus or the twitch. Half relaxation time (*hrt*): time for the transient to return to 50% of the peak during the relaxation phase. Shortening velocity (departure velocity, *v*_*dep*_): characterizes the maximum speed with which the cell contracts. Twitch amplitude (peak h): difference between the peak and baseline values. *n* = number of analyzed single cells in at least two differentiations (*N* ≥ 2). Differentiations were performed using one isogenic control clone and two heterozygous *MYBPC3* mutant clones. Data are presented as mean ± SD. Statistical comparisons were analyzed by Mann-Whitney test

At d35 twitch time to peak (*ttp)*, half relaxation time *(hrt)*, and maximum shortening velocity (*v*_*dep*_) were similar between mutant and control CMs (Fig. 5A). However, after 56 days of cultivation, *MYBPC3*^c.927–2 A> G^ hiPSC-CMs exhibited increased *ttp* and *hrt*, and reduced amplitude of twitch contraction (Fig. 5B). This aligns with reduced *v*_*dep*_ for CMs at d56 altogether indicating slower twitch kinetics (Fig. 5B). By d70+, *ttp* and *hrt* were no longer significantly different between genotypes. At this time point, the isogenic control CMs exhibited a slight reduction in twitch amplitude and *v*_*dep*_, which may reduce genotype separation for *ttp* and *hrt*. Importantly, mutation-associated functional deficits persisted at d70+, as twitch amplitude and *v*_*dep*_ of the mutant CMs remained reduced when compared to the isogenic control (Fig. 5C).

For the analysis of twitch kinetics, contractions of both, single cardiomyocytes (SCM) and connected cardiomyocytes or cardiomyocytes within clusters were recorded. To find out whether twitch kinetics differ between SCM and clusters we analyzed the recordings separately. Given that the most pronounced difference in *v*_*dep*_ was observed at day 70+, we focused on the results from this time point. Notably, the *v*_*dep*_ of SCM at day 70 + was comparable between the isogenic control and the mutant (Supplemental Fig. 5A). However, when analyzing only connected CMs, a greater reduction in *v*_*dep*_ was observed for the mutant (Supplemental Fig. 5B). This may indicate that the primary difference in *v*_*dep*_ arises from the slower twitch kinetics of mutant CMs within the clusters.

Given the observed reduction in shortening velocity, we further examined calcium transient parameters to assess whether alterations in Ca²⁺ handling contribute to the observed changes in contraction kinetics. Calcium transients were recorded at 1 Hz/35 V stimulation and 37 °C following the loading of hiPSC-CMs with the intracellular calcium ratiometric indicator Fura-2-AM.

**Fig. 6:**
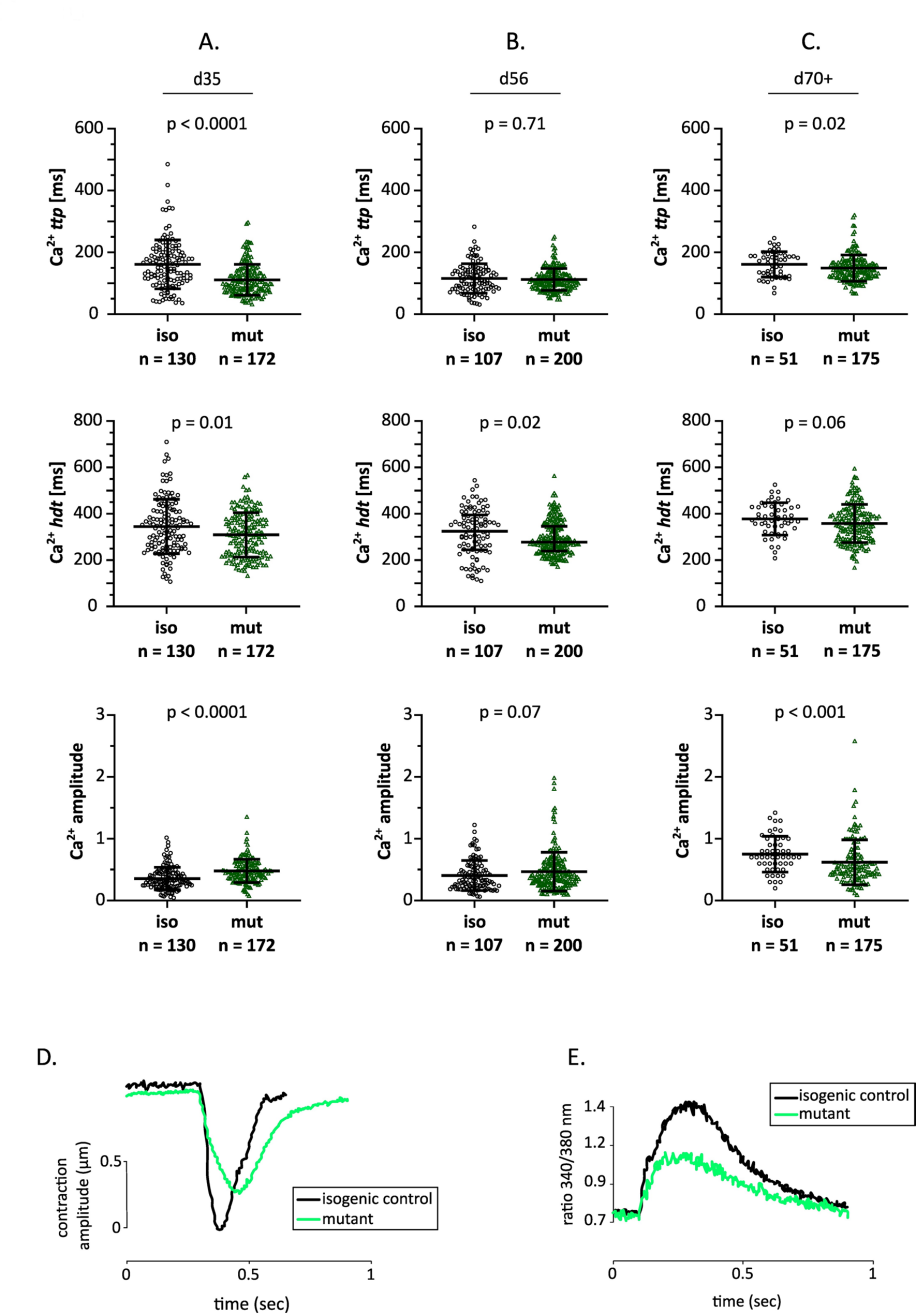
Scatter plots of Ca^2+^-transient parameters of *MYBPC3*^c.927–2 A> G^ and isogenic control hiPSC-CMs. Ca^2+^ time to peak (Ca^2+^-*ttp*): the time at which the Ca^2+^ peak is reached starting from the electrical stimulus for the twitch. Ca^2+^ half decay time (Ca^2+^-*hdt*): time for Ca^2+^ transient to reach to 50% of the peak during the Ca^2+^ reuptake phase. Ca^2+^ amplitude: difference between the peak and baseline values (340/380 nm ratio). *n* = number of analyzed single cells in three differentiations (*N* = 3). Differentiations were performed using one isogenic control clone and two heterozygous *MYBPC3* mutant clones. Statistical comparisons were analyzed using Mann-Whitney test. **D**, **E** Representative twitch (**D**) and Ca^2+^ transient (**E**) recordings from d70 + of cultivation for isogenic control and mutant *MYBPC3*^c.927–2 A> G^ hiPSC-CM

We observed reduced Ca^2+^-transient time to peak (Ca^2+^-*ttp*) and half-decay time (Ca^2+^-*hdt*) of mutant CMs compared to isogenic control CMs at d35 (Fig. 6A). Concurrently, the Ca^2+^ transient amplitude was elevated in the *MYBPC3*^c.927–2 A> G^ hiPSC-CMs. This phenotype became less pronounced by d56, with comparable Ca^2+^ transient parameters between the isogenic control and the mutant (Fig. 6B). However, at d70+, the Ca^2+^ amplitude of mutant CMs decreased compared to isogenic control CMs, accompanied by slightly shorter, Ca^2+^-*ttp* and Ca^2+^-*hdt* (Fig. 6C). Overall, the Ca^2+^ transient of mutant CMs initially was faster, followed by changes in the opposite direction over the cultivation period for the mutant compared to isogenic control.

### mRNA Sequencing and Gene Set Enrichment analysis

**Fig. 7:**
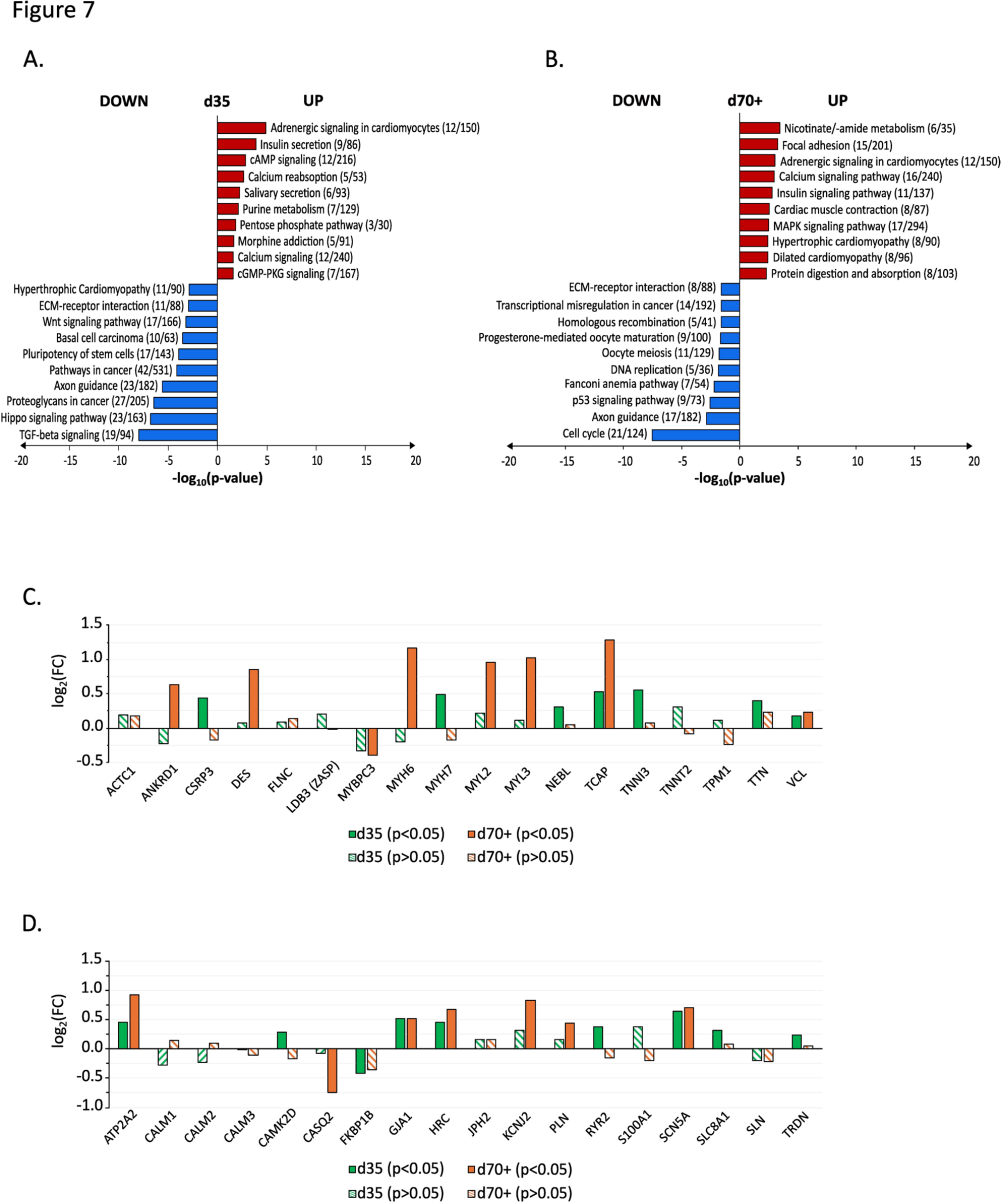
Top 10 of dysregulated pathways and proteins associated with the *MYBPC3*^c.927–2 A> G^ mutation. **A**, **B** The HCM-associated pathways in *MYBPC3*^c.927–2 A> G^ hiPSC-CMs identified through enrichment analysis (KEGG database) at day 35 (**A**) and day 70+ (**B**). Each bar represents a specific pathway, with its length corresponding to the degree of statistical significance (log₁₀(p-value)). **C**,** D** Bar chart showing log₂ fold change (log₂FC) values of dysregulated genes in mutant hiPSC-CMs compared to isogenic controls. Dysregulated genes are grouped into two categories: sarcomeric genes (**C**) and calcium handling genes (**D**). Green bars represent genes at day 35; orange bars represent genes at day 70+. Bars with a line pattern indicate changes that were not statistically significant (*p* > 0.05)

To investigate the dysregulated genes and pathways associated with the *MYBPC3* c.927–2 A > G mutation, mRNA sequencing was performed on d35 and d70+, comparing mutant CMs with isogenic controls. Among the top ten enriched pathways at d35, significant enrichment in the downregulated fraction was observed in HCM pathway (Fig. 7A). In contrast, at d70+, HCM, dilated cardiomyopathy, calcium signalling, and cardiac muscle contraction pathways were significantly enriched in the upregulated fraction (Fig. 7B).

Subsequently, gene clusters associated with sarcomere contraction, assembly, and calcium handling were analyzed. The most pronounced differences in log₂ fold-change (log₂FC) of dysregulated genes were observed at d70+, while changes at day 35 were less pronounced. mRNA sequencing highlighted the upregulation of *MYL2*,* MYH6*, and *MYL3* at d70+, genes associated with the enrichment of HCM and cardiac muscle contraction pathways (Fig. 7C). Notably, these genes were not significantly dysregulated in the mutant at d35 (Fig. 7C).

Similarly, genes crucial for sarcomere assembly and maintenance, such as *TCAP* and *DES*, were significantly upregulated at d70+, but showed minimal changes at d35 (Fig. 7C). Genes involved in calcium handling and cellular excitation exhibited marked dysregulation at d70+, with upregulation of *ATP2A2*,* HRC*,* KCNJ2*, and *SCN5A*, while *CASQ2* was downregulated (Fig. 7D). These findings suggest an alteration in gene expression associated with the *MYBPC3* c.927–2 A > G mutation at the later time point, particularly affecting pathways critical for cardiomyocyte function.

## Discussion

In this study, we aimed to elucidate the impact of HCM-related *MYBPC3* c.927–2 A > G mutation on hiPSC-CMs function and to characterize the phenotype development in a time-dependent manner. We performed extensive, long-term analyses (70 + days) with high replicate numbers in *MYBPC3* c.927–2 A > G and isogenic control hiPSC-CMs. For most experiments, we used one heterozygous mutant clone and one isogenic control clone, which may not fully exclude clonal variability and limits generalizability of the findings. Yet, all clones were derived under identical conditions and underwent comprehensive characterization including karyotyping, flow cytometry, and genomic stability.

While cMyBP-C haploinsufficiency has been reported across multiple model systems and patient samples, the magnitude of reduction in bulk cMyBP-C levels in 2D hiPSC-CM studies is not uniform [14, 16, 30–31]. Indeed, several reports have described preserved or even increased cMyBP-C levels in *MYBPC3*-mutant 2D hiPSC-CMs [14, 33, 34], highlighting that the causal variant, maturation state, culture format, and protein homeostasis/turnover can substantially influence the expected reduction in cMyBP-C levels. These observations suggest that relying solely on bulk protein analysis (such as Western blot) to define haploinsufficiency may underestimate other mutation-associated effects on a protein expression. Accordingly, the major focus of this study was the mode of *MYBPC3* transcription and the variability of wild-type cMyBP-C expression, which may be linked to the mosaic-like pattern shown in patient tissue by us and others [16, 35]. Importantly, a higher proportion of cMyBP-C negative cells was observed in our cultures and supports the mosaic hypothesis. Future studies applying more quantitative approaches, such as capillary immunoassays or targeted mass spectrometry, will be valuable to further refine absolute cMyBP-C quantification across maturation stages and culture formats.

Our findings confirmed cMyBP-C haploinsufficiency in mutant hiPSC-CMs at day 35 and 70 + but not at day 56. The observations in our study suggest that at d56, NMD of truncated *MYBPC3*-mRNA in mutant CMs [17] may be compensated for at the protein level. This could well be linked to increased transcriptional activity in response to cMyBP-C haploinsufficiency. RNA-FISH showed overall more *MYBPC3* aTS per nucleus in mutant CMs, and higher transcriptional activity for both mutant and isogenic control CMs at d56. Considering the potentially slower degradation of cMyBP-C in *MYBPC3* mutant CMs reported by Helms et al. [14], elevated transcription could temporarily normalize protein levels at d56. Nevertheless, immunofluorescence revealed an increasing number of cMyBP-C-negative cells, indicating that transcriptional upregulation is insufficient to prevent variable cMyBP-C expression over time. This aligns with observations in symptomatic HCM patients, where increased transcriptional activity, but variable *MYBPC3* mRNA and cMyBP-C protein levels from CM to CM were found [17].

Important to note, cardiomyocyte nuclei can exhibit > 2 aTS due to increased ploidy (e.g., endoreduplication / polyploidization), which is common in this cell type [36] and has also been shown in myocardial tissue [27]. It should be noted that both, diploid and polyploid nuclei, contribute to unequal allelic expression and occur in mutant and isogenic hiPSC-CMs. Thus, our conclusions do not rely on assigning biological meaning to individual nuclei with > 2 aTS; rather, they are based on comparative trends and distributional differences between mutant and isogenic control populations across time points and independent differentiations.

The observed increase in variable expression of cMyBP-C from CM to CM in mutant hiPSC-CMs may result from burst-like *MYBPC3* transcription. Given that each active transcription site produces either wild type protein or, if the mutant allele is transcribed, no detectable protein, cardiomyocytes lacking cMyBP-C are likely cells in which transcription predominantly occurred from the mutant allele at that time. In contrast to observations in patient cardiac tissue [16], we also identified cMyBP-C-negative cells in isogenic controls, with their proportion increasing over extended culture periods. If cMyBP-C degradation occurs more rapidly in isogenic controls than in mutants [14], this could contribute to the observed increase in cMyBP-C-negative cells. The presence of cMyBP-C-negative cells in the isogenic control population may indicate biological heterogeneity in this hiPSC-CM system, potentially reflecting early apoptotic CM states or variability related to burst-like transcription and protein turnover at later time points. Nonetheless, the proportion of cMyBP-C-negative cells in the mutant remained consistently twice as high as in controls, reaching 40% by day 70+, supporting a mutation-associated effect beyond heterogeneity level observed in controls.

Apart from variable cMyBP-C expression, mutant hiPSC-CMs exhibited disrupted cardiomyocyte morphology, characterized by increased myofibrillar disarray and a loss of elongated cell shape. This could be due to unequal cMyBP-C expression from CM to CM resulting in unequal contraction, depending on proper cMyBP-C stoichiometry within the sarcomeres. In addition, the disorganization of myofibrils suggests that cMyBP-C deficiency may also directly compromise sarcomere integrity and alignment. Transcriptomic analysis revealed upregulation of *TCAP* (titin-cap) and *DES* (desmin), both of which are associated with the Z-disc and play critical roles in sarcomere organization, sarcomere-membrane interaction, and mechanical stretch sensing [37–41]. The observed increase in *TCAP* and *DES* expression, particularly at later time points of cultivation (day 70+), may reflect an adaptive response to structural disarray and the associated alterations in mechanical tension.

Different from prior hiPSC-CM studies of *MYBPC3* mutations [42, 43] we did not observe significant changes in cardiomyocyte cell area. This discrepancy may be attributable to differences in experimental conditions, particularly the use of nanopatterned substrates in our study. It has been demonstrated that specific nanoscale topographies, such as ridges and grooves, promote cardiomyocyte elongation but restrict width growth compared to unpatterned surfaces [44, 45]. This geometric influence might preserve to some extent the organized structure characteristic of healthy myocardium and physically limit hypertrophic cell expansion by spatially confining cytoskeletal assembly and focal adhesions [44]. Previous HCM studies employed flat substrates that allow unrestricted spreading, which may make cell area differences more pronounced.

Since cMyBP-C deficiency has been linked to impaired cardiac function, we assessed twitch contraction parameters and calcium transients. Over time, mutant hiPSC-CMs exhibited a decline in contraction velocity, which may also be attributed to an increase in myofibrillar disarray, possibly correlated with an unequal intracellular distribution of cMyBP-C. Disorganized myofibrils likely impair sarcomere function, leading to reduced shortening velocity.

Interestingly, by day 70+, difference in contraction velocity between isogenic control and mutant CMs was primarily observed in hiPSC-CMs recorded within clusters, whereas single cardiomyocytes did not show significant differences to isogenic controls. Due to technical limitations, we could not directly correlate cMyBP-C levels with contraction kinetics at the single-cell level. Nevertheless, the observed reduction in contraction velocity in mutant cardiomyocytes needs to be further verified within multicellular clusters or bioengineered cardiac tissues [21].

While twitch shortening velocity slowed over cultivation time in mutant hiPSC-CMs, Ca²⁺ handling exhibited accelerated dynamics, and an initial increase in Ca²⁺ transient amplitude at day 35 followed by a decrease at day 70+. The most pronounced differences in Ca²⁺ transients between mutants and controls were found at day 35, while twitch kinetics remained similar. Transcriptomic analysis revealed an upregulation of *ATP2A2* (SERCA), which could enhance Ca²⁺ reuptake into the sarcoplasmic reticulum (SR), and *HRC* (histidine-rich Ca^2+^ binding protein), which may increase SR Ca²⁺ storage capacity and facilitate faster Ca^2+^ release [45,46]. These changes align with the shorter Ca²⁺ time-to-peak and half-decay times of Ca²⁺ transients from mutant CMs.

In *MYBPC3*-related HCM, impaired cardiomyocyte function can be strongly influenced by myofilament-level alterations, including changes in myofilament Ca²⁺ sensitivity and/or cross-bridge kinetics. Thus, faster cytosolic Ca²⁺ decline may not be sufficient to normalize twitch kinetics.

In this study, accelerated Ca²⁺ handling in mutant CMs may therefore represent a potentially compensatory adjustment in Ca^2+^ handling that partially counterbalances impaired contractile function. Other *MYBPC3*-HCM studies report divergent Ca²⁺ phenotypes, including faster or preserved Ca²⁺ decay despite impaired relaxation/diastolic function [47, 48], prolonged Ca²⁺ transients in some human samples [32], and distinct Ca²⁺ handling alterations in engineered tissue models that do not necessarily map directly onto contraction kinetics [33]. Differences in mutation type, culture format (animal, 2D monolayer, engineered heart tissue), maturation state, pacing, load conditions, and analysis time point could potentially contribute to this variability. Accordingly, our data most likely reflect an early-stage phenotype in which slower twitch kinetics coexist with a potentially compensatory acceleration of Ca²⁺ removal under our specific culture conditions and time points, and therefore should not be interpreted as a universal *MYBPC3*-HCM Ca²⁺-handling signature.

In conclusion, our patient-specific hiPSC-CMs model robustly recapitulates key aspects of the *MYBPC3* c.927–2 A > G HCM phenotype in a time-dependent manner, including cMyBP-C haploinsufficiency, *MYBPC3* burst-like transcription and variable cMyBP-C expression from CM to CM. This model demonstrates that variability in cMyBP-C expression may play an important role in disease pathogenesis and provides a valuable platform to further investigate HCM development and progression.

## Supplementary Information

Below is the link to the electronic supplementary material.

Supplementary Material 1: Fig. 1 Sequencing validation of MYBPC3 in isogenic control and heterozygous MYBPC3 mutant hiPSC lines. Sanger sequencing of the MYBPC3 gene region used to confirm the genotypes of the isogenic control and heterozygous MYBPC3 mutant hiPSC-lines. A: The isogenic control line carries a homozygous silent mutation. B: The heterozygous mutant line carries the same silent mutation on one allele, and a pathogenic missense mutation (c.927–2 A > G) on the other allele. Sequencing chromatograms illustrate the presence of a single peak in the isogenic control and dual peaks at the mutation site in the heterozygous mutant, confirming successful genome editing. C: Karyograms obtained for the isogenic control, heterozygous mutant clone 1 and clone 2 revealed no chromosomal abnormalities.

Supplementary Material 2: Fig. 2 Flow cytometry analysis of PFA-fixed, permeabilized hiPSCs against the pluripotency markers NANOG, OCT-3/4, SSEA-3 and TRA-1-60 showed > 90% pluripotent cells.

Supplementary Material 3: Fig. 3 Genomic stability was assessed by detection of recurrent genetic abnormalities using the iCS-digital™ PSC assay (Stem Genomics; [18]). All lines carried a common 20q11.21 gain, reported to affect neuroectodermal but not mesodermal differentiation [50]. Cardiomyocyte differentiation was not impacted in our experiments, as confirmed by flow cytometry. No other abnormalities were detected.

Supplementary Material 4: Fig. 4 Western blot images showing protein expression of cMyBP-C and α-actinin at d35 (A), d56 (B), and d70+ (C). Samples were loaded from three separate differentiation batches, indicated in the sample names by a combination of the cell line ID and the differentiation number (e.g., “mut 2” = mutant, second differentiation). The empty lanes (technical issue e.g., low material/transfer artifact, marked with *) were excluded from densitometric analysis. Exposure time: 30 s. (D) Original full-length uncropped blots.

Supplementary Material 5: Fig. 5 Shortening velocity of single vs. cluster-connected hiPSC-CMs after 70 + days of cultivation. Data include recordings from individual isolated cardiomyocytes (A) and from cardiomyocytes located at the edge or within cellular clusters (B). This comparison highlights potential functional differences related to cell-cell interactions.

Supplementary Material 6: Fig. 6 Principal component analysis (PCA) of normalized RNA-seq counts (Phantasus Web Application) across independent differentiations to assess potential batch effects. (A) Day 35 samples are separated by genotype (primarily along PC1). Within the isogenic control group one differentiation deviates from the main cluster. (B) Day 70 + samples of isogenic control and MYBPC3 mutant are separated along PC1.

Supplementary Material 7: Table 1 RNA-seq differential expression analysis in MYBPC3 c.927–2 A > G and isogenic control hiPSC-CMs at day 35 and day 70+. Table reports DESeq2 size-factor-normalized read counts for each gene (per sample/replicate), together with DESeq2 differential expression results for mutant versus isogenic control at day 35 and day 70+ [log2 fold change, Wald test p value, and Benjamini-Hochberg FDR-adjusted p value (padj)]. Genes are sorted by padj. Results were estimated by the DESeq2 negative binomial generalized linear model on normalized counts.

## Data Availability

Data is provided within the manuscript or supplementary information files.
