# Supplementary figures and images for: Variable cMyBP-C expression from cell to cell in a MYBPC3c.927–2 A>G hiPSC-CM model recapitulates HCM patient phenotype
Source: Stem Cell Res Ther. 2026 May 19;17:188. doi: 10.1186/s13287-026-05063-9 (PMC13185243; doi:10.1186/s13287-026-05063-9)

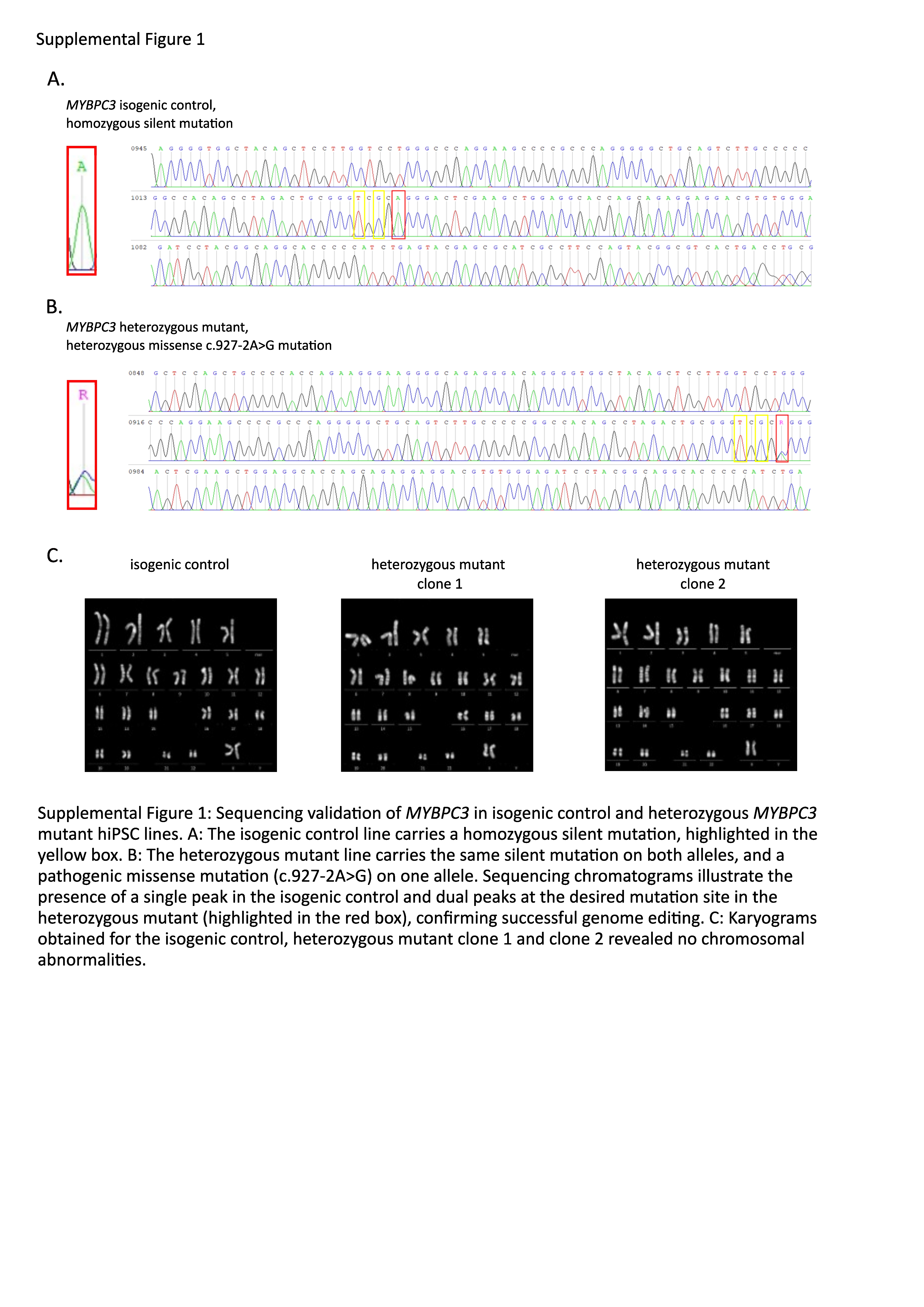

Supplement: Supplementary file 1 — Supplementary Material 1: Fig. 1 Sequencing validation of MYBPC3 in isogenic control and heterozygous MYBPC3 mutant hiPSC lines. Sanger sequencing of the MYBPC3 gene region used to confirm the genotypes of the isogenic control and heterozygous MYBPC3 mutant hiPSC-lines. A: The isogenic control line carries a homozygous silent mutation. B: The heterozygous mutant line carries the same silent mutation on one allele, and a pathogenic missense mutation (c.927–2 A > G) on the other allele. Sequencing chromatograms illustrate the presence of a single peak in the isogenic control and dual peaks at the mutation site in the heterozygous mutant, confirming successful genome editing. C: Karyograms obtained for the isogenic control, heterozygous mutant clone 1 and clone 2 revealed no chromosomal abnormalities. [file 13287_2026_5063_MOESM1_ESM.png]

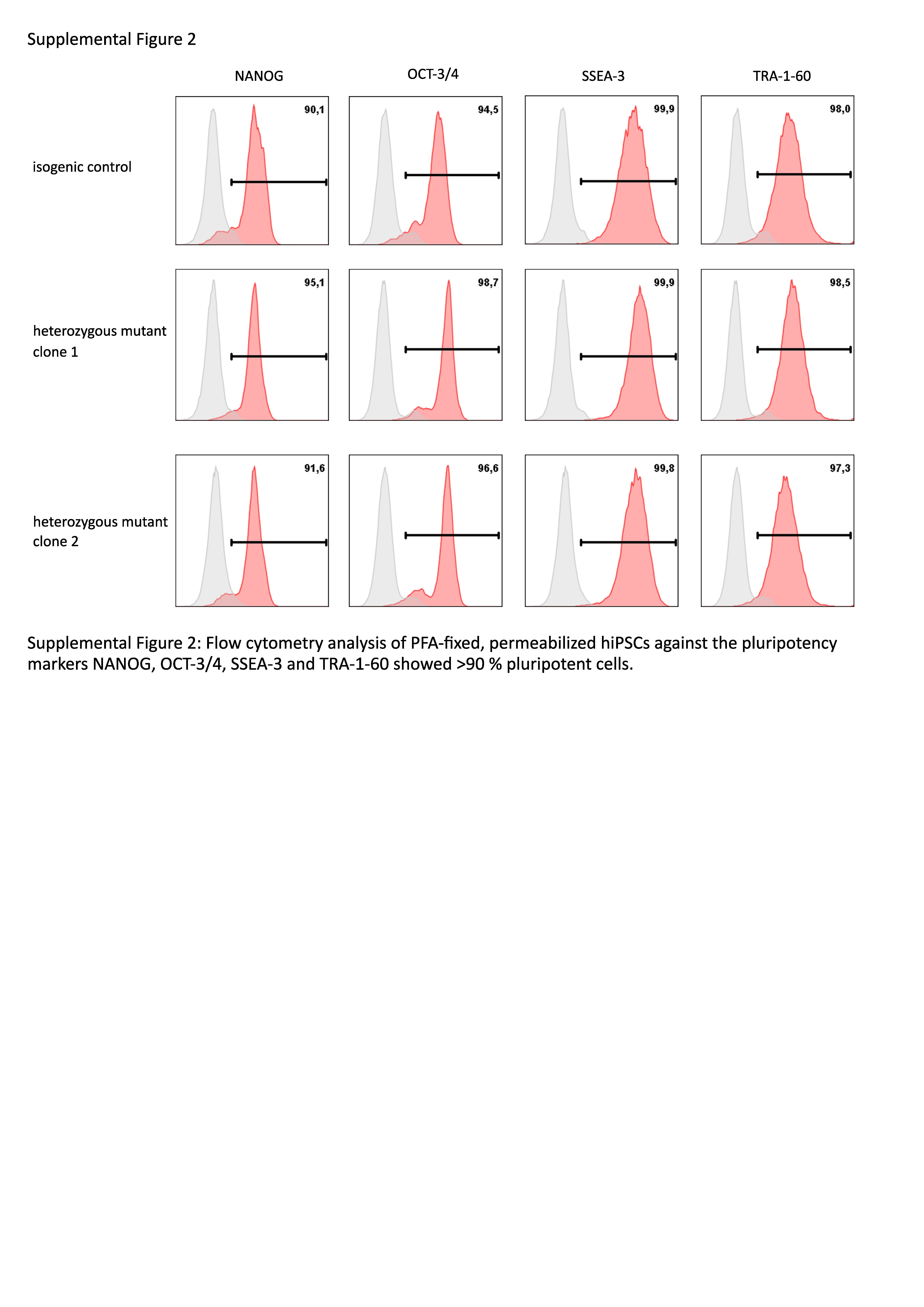

Supplement: Supplementary file 2 — Supplementary Material 2: Fig. 2 Flow cytometry analysis of PFA-fixed, permeabilized hiPSCs against the pluripotency markers NANOG, OCT-3/4, SSEA-3 and TRA-1-60 showed > 90% pluripotent cells. [file 13287_2026_5063_MOESM2_ESM.jpg]

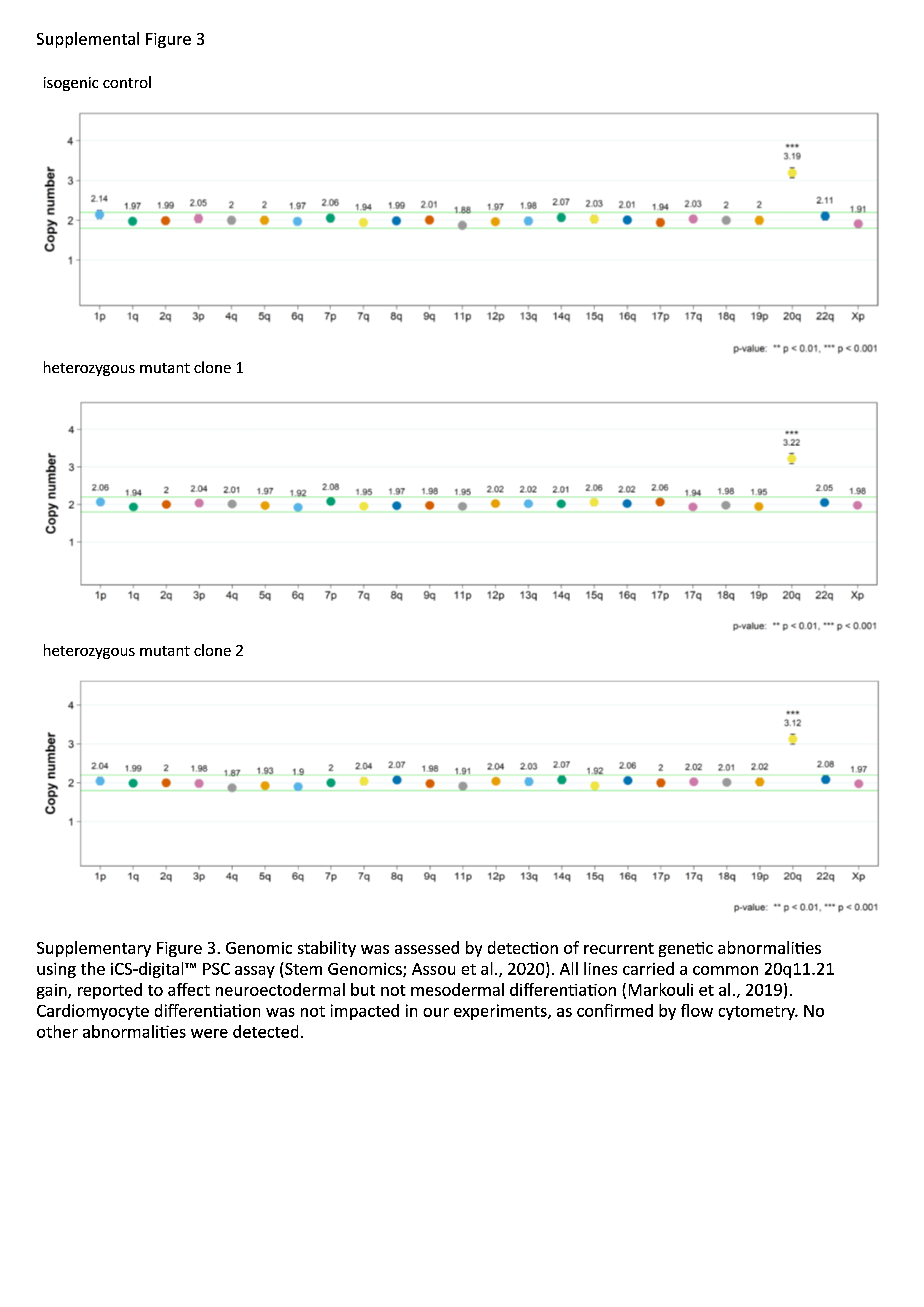

Supplement: Supplementary file 3 — Supplementary Material 3: Fig. 3 Genomic stability was assessed by detection of recurrent genetic abnormalities using the iCS-digital™ PSC assay (Stem Genomics; [18]). All lines carried a common 20q11.21 gain, reported to affect neuroectodermal but not mesodermal differentiation [50]. Cardiomyocyte differentiation was not impacted in our experiments, as confirmed by flow cytometry. No other abnormalities were detected. [file 13287_2026_5063_MOESM3_ESM.jpg]

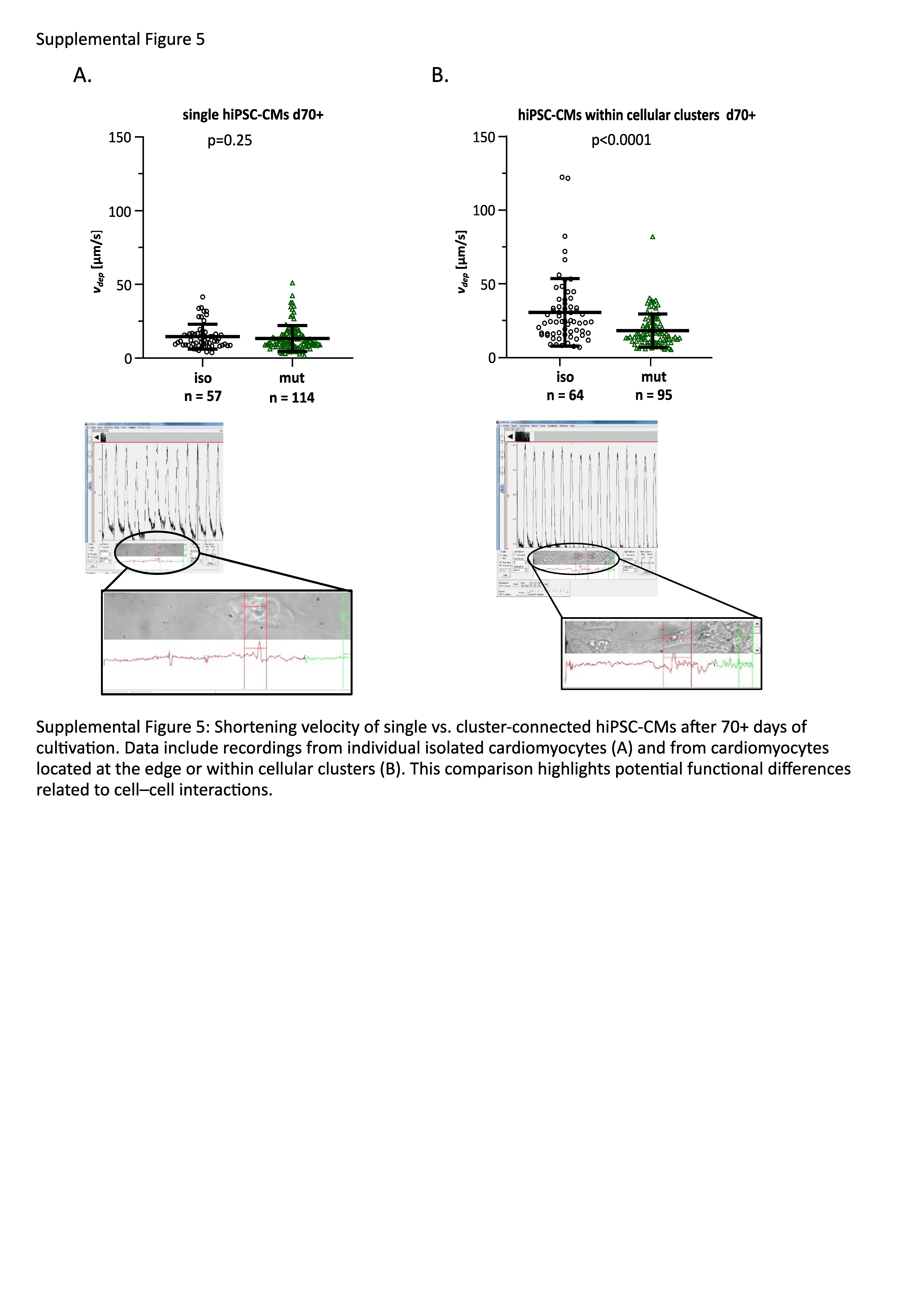

Supplement: Supplementary file 4 — Supplementary Material 4: Fig. 4 Western blot images showing protein expression of cMyBP-C and α-actinin at d35 (A), d56 (B), and d70+ (C). Samples were loaded from three separate differentiation batches, indicated in the sample names by a combination of the cell line ID and the differentiation number (e.g., “mut 2” = mutant, second differentiation). The empty lanes (technical issue e.g., low material/transfer artifact, marked with *) were excluded from densitometric analysis. Exposure time: 30 s. (D) Original full-length uncropped blots. [file 13287_2026_5063_MOESM4_ESM.jpg]

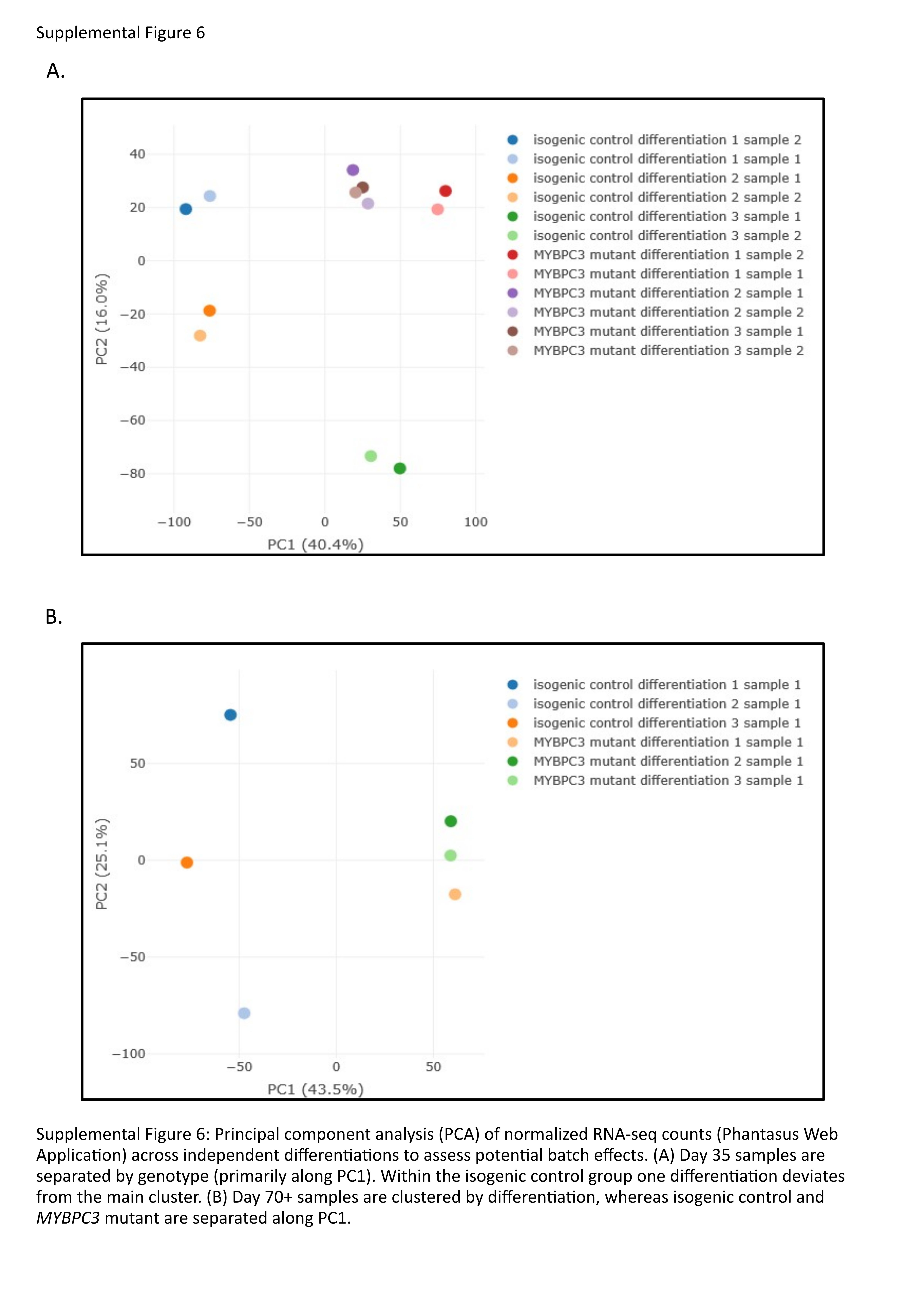

Supplement: Supplementary file 5 — Supplementary Material 5: Fig. 5 Shortening velocity of single vs. cluster-connected hiPSC-CMs after 70 + days of cultivation. Data include recordings from individual isolated cardiomyocytes (A) and from cardiomyocytes located at the edge or within cellular clusters (B). This comparison highlights potential functional differences related to cell-cell interactions. [file 13287_2026_5063_MOESM5_ESM.jpg]
