# Supplementary material for: Variable cMyBP-C expression from cell to cell in a MYBPC3c.927–2 A>G hiPSC-CM model recapitulates HCM patient phenotype
Source: Stem Cell Res Ther. 2026 May 19;17:188. doi: 10.1186/s13287-026-05063-9 (PMC13185243; doi:10.1186/s13287-026-05063-9)

Supplemental Figure 4 A-C

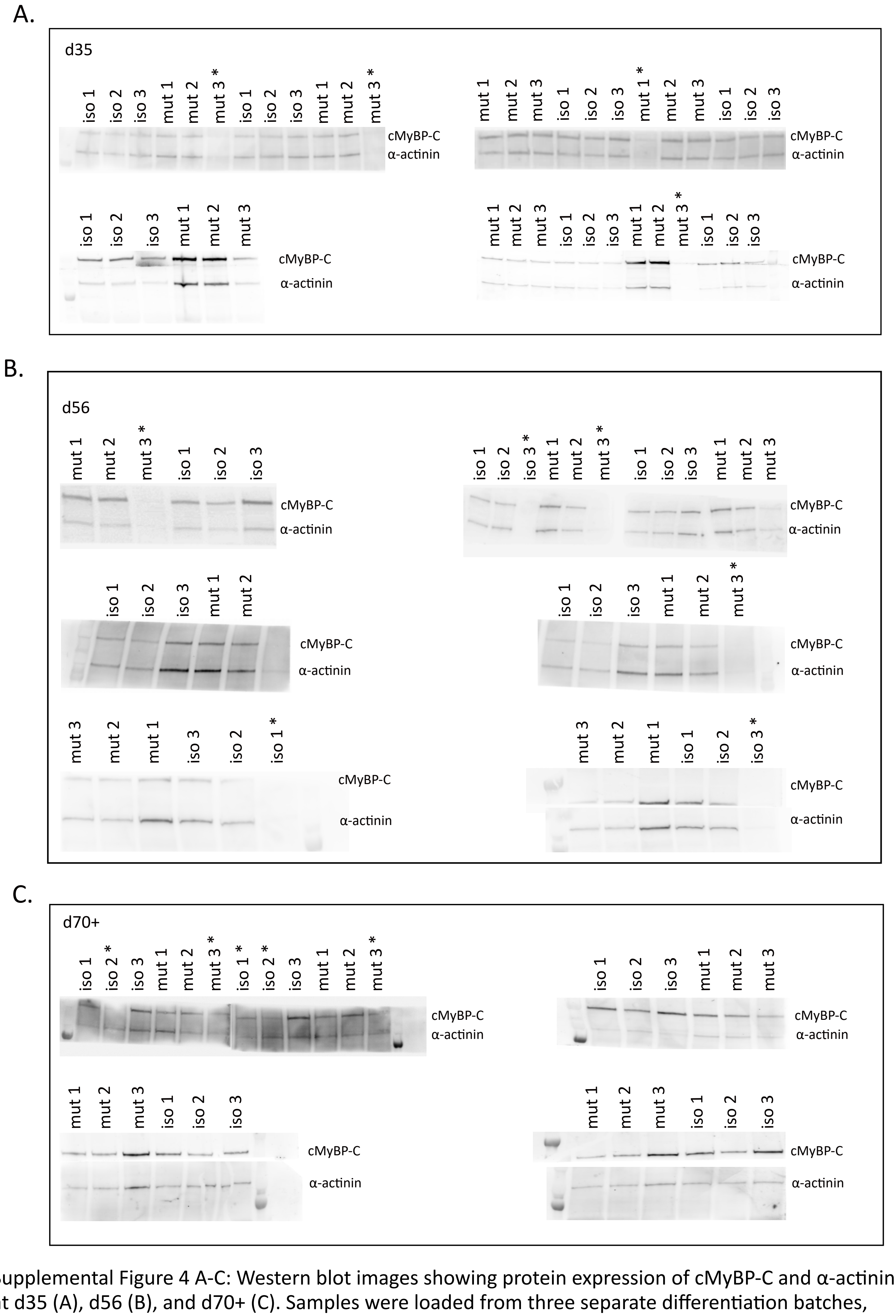


Supplemental Figure 4 A-C: Western blot images showing protein expression of cMyBP-C and α-actinin at d35 (A), d56 (B), and d70+ (C). Samples were loaded from three separate differentiation batches, indicated in the sample names by a combination of the cell line ID and the differentiation number (e.g., "mut 2"" = mutant, second differentiation). The empty lanes (technical issue e.g., low material/ transfer artifact, marked with *) were excluded from densitometric analysis. Exposure time: 30 seconds.

Supplemental Figure 4 D: To optimize antibody usage and enable simultaneous detection of target proteins, PVDF membranes were stained with Ponceau S and horizontally sectioned according to molecular weight markers prior to antibody incubation. Alpha-actinin and cMyBP-C were detected on the same membrane segment, corresponding approximately to the 75-160 kDa range.  All full-membrane images corresponding to Supplementary Figure 4 A-C are provided. Where available, images of the cut membranes are also included. In cases where no image of the cut membrane is provided, black marks indicate the positions at which the membrane was cut prior to antibody incubation.

Full PVDF membrane images and corresponding Western Blot images for day 35 (S. 4A):

Gel1:

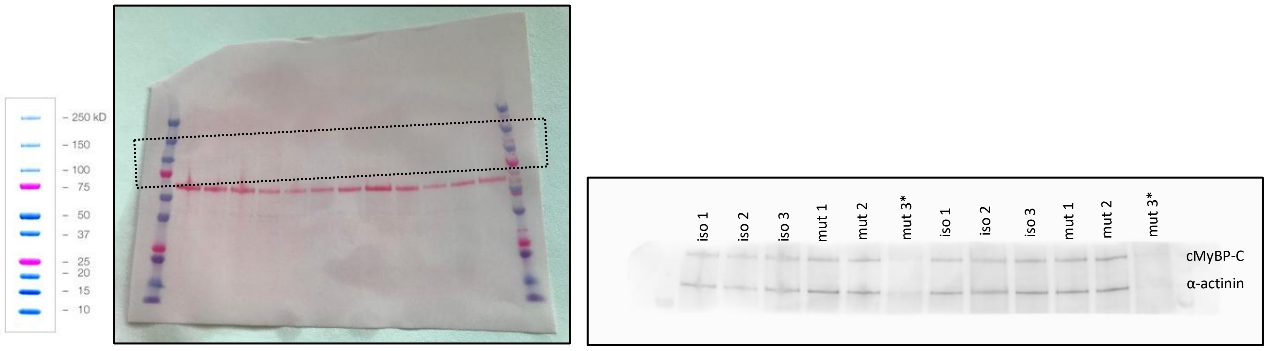


Gel2:

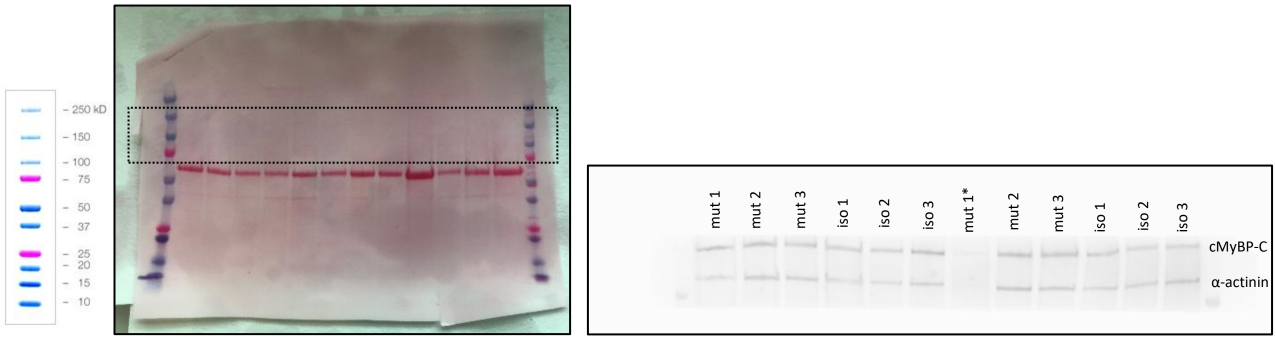


Gel3:

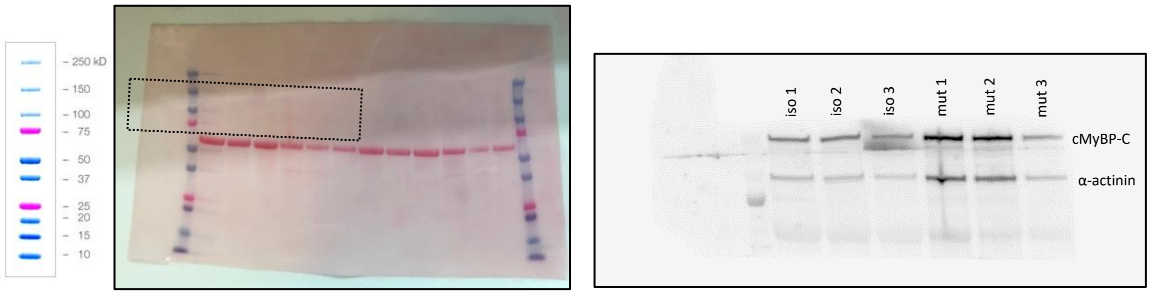


Gel4:

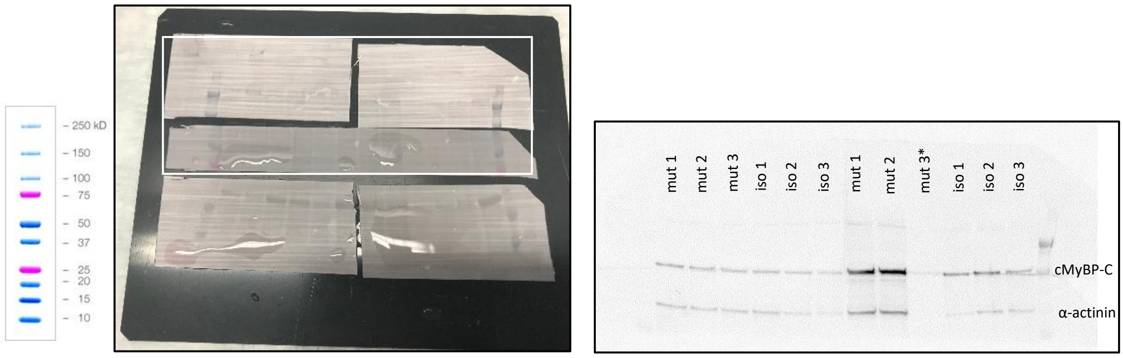

Full PVDF membrane images and corresponding Western Blot images for day 56 (S. 4B):

Gel1:

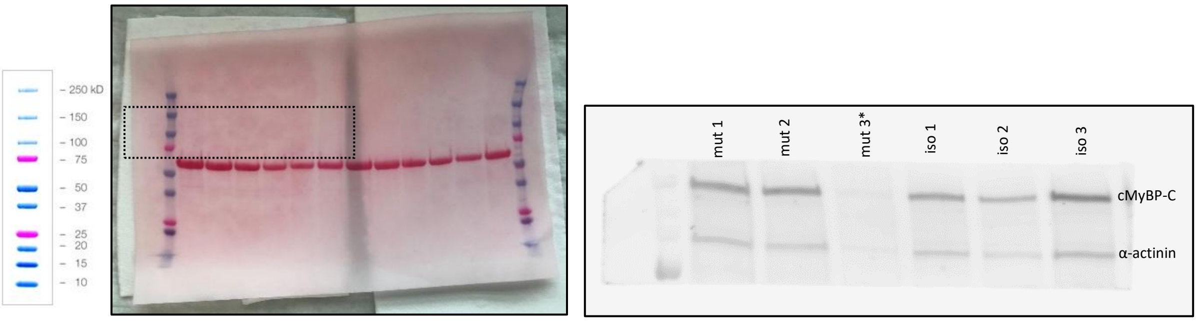


Gel2:

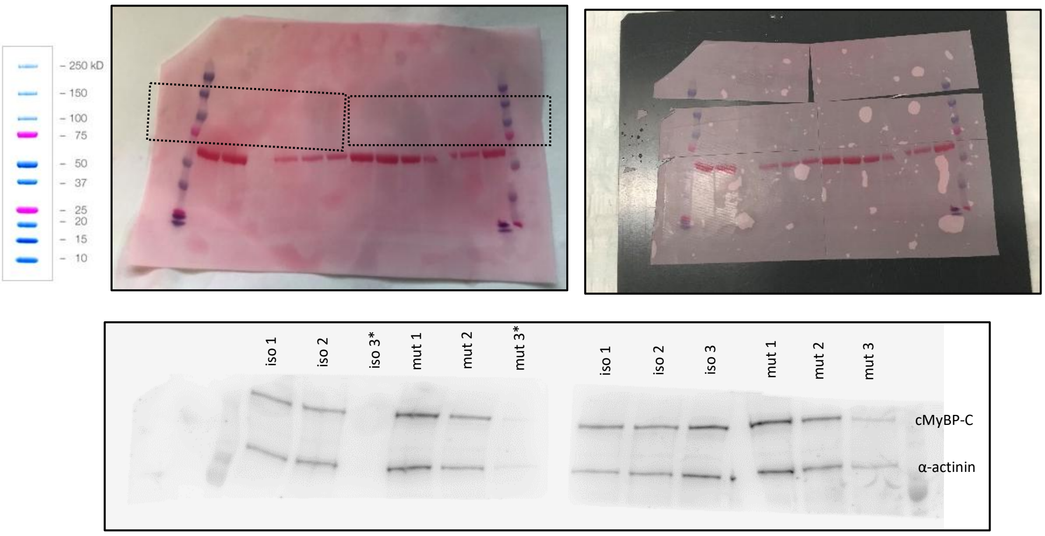


Gel3:

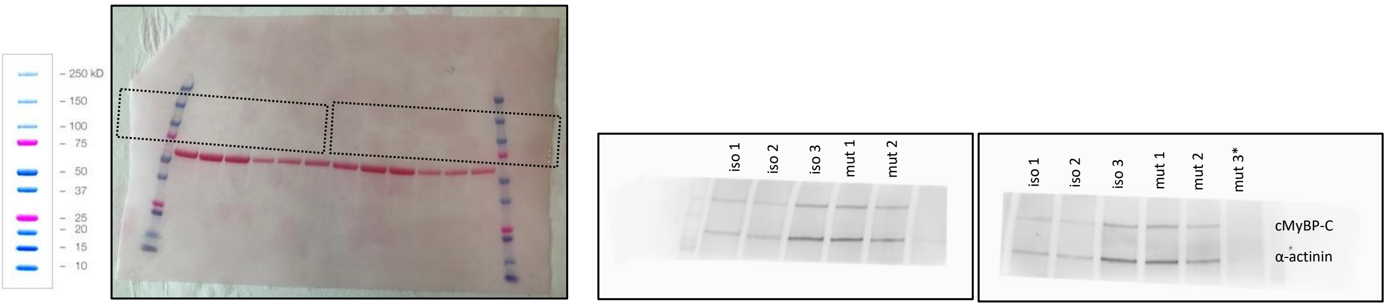


Gel4:

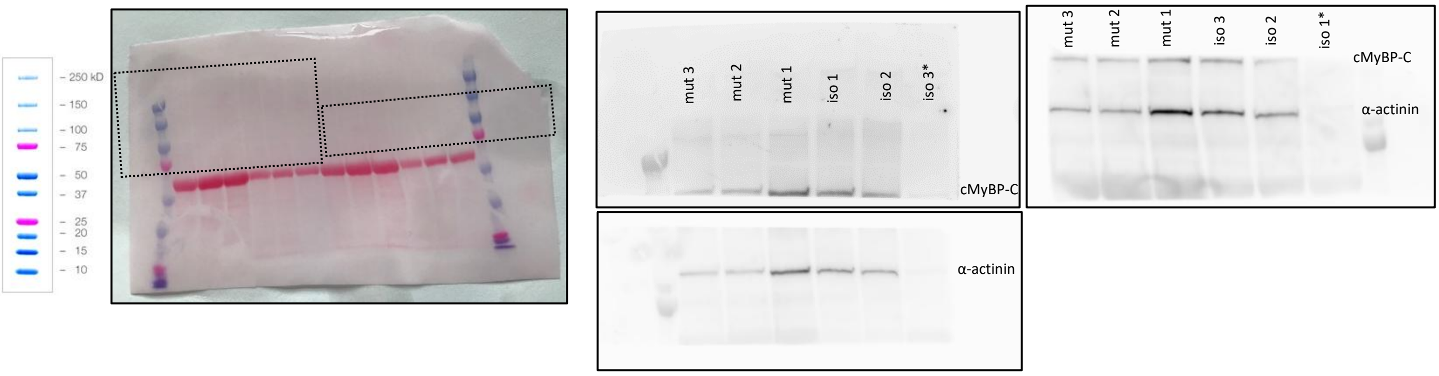

Full PVDF membrane images and corresponding Western Blot images for day 70+ (S. 4C):

Gel1:

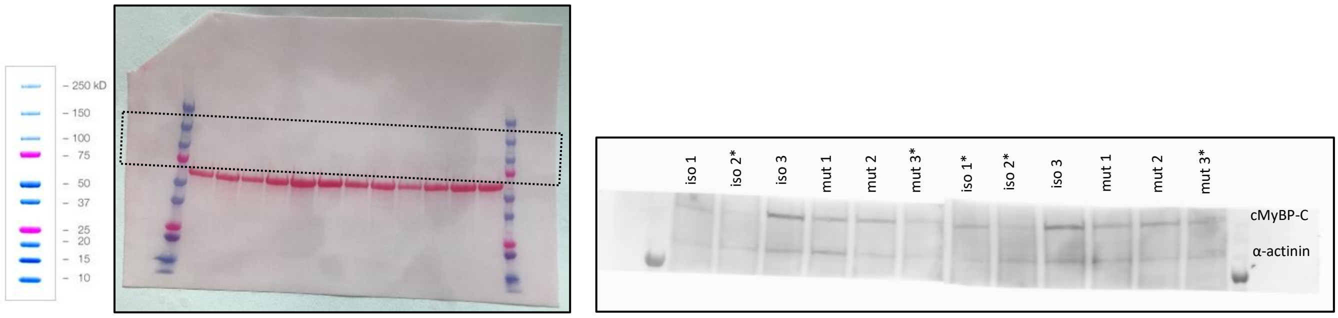


Gel2:

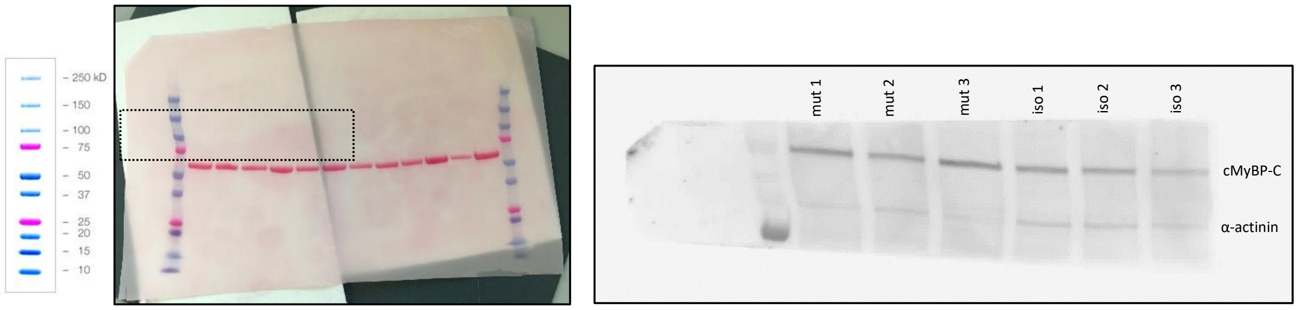


Gel3:

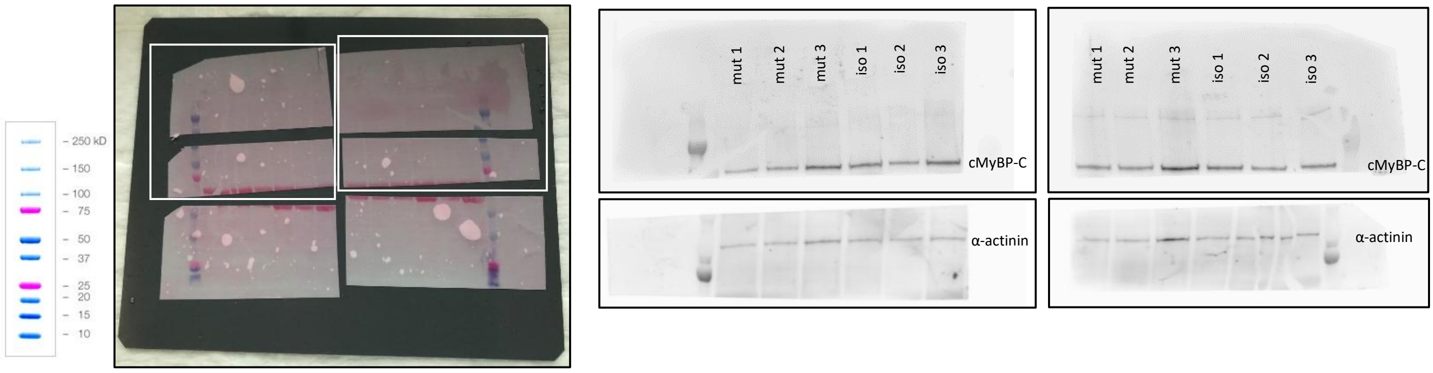

Supplement: Supplementary file 7 — Supplementary Material 7: Table 1 RNA-seq differential expression analysis in MYBPC3 c.927–2 A > G and isogenic control hiPSC-CMs at day 35 and day 70+. Table reports DESeq2 size-factor-normalized read counts for each gene (per sample/replicate), together with DESeq2 differential expression results for mutant versus isogenic control at day 35 and day 70+ [log2 fold change, Wald test p value, and Benjamini-Hochberg FDR-adjusted p value (padj)]. Genes are sorted by padj. Results were estimated by the DESeq2 negative binomial generalized linear model on normalized counts. [file 13287_2026_5063_MOESM7_ESM.pdf]
